# Estimating the proportion of guilty suspects and posterior probability of guilt in lineups using signal-detection models

**DOI:** 10.1186/s41235-020-00219-4

**Published:** 2020-05-13

**Authors:** Andrew L. Cohen, Jeffrey J. Starns, Caren M. Rotello, Andrea M. Cataldo

**Affiliations:** 1 Department of Psychological and Brain Sciences, University of Massachusetts, Amherst, MA 01003 USA; 2 McLean Hospital, Harvard Medical School, Belmont, MA USA

**Keywords:** Eyewitness lineup, Confidence, Signal detection theory, Computational modeling, Posterior probability of guilt, Base rate

## Abstract

**Background:**

The majority of eyewitness lineup studies are laboratory-based. How well the conclusions of these studies, including the relationship between confidence and accuracy, generalize to real-world police lineups is an open question. Signal detection theory (SDT) has emerged as a powerful framework for analyzing lineups that allows comparison of witnesses’ memory accuracy under different types of identification procedures. Because the guilt or innocence of a real-world suspect is generally not known, however, it is further unknown precisely how the identification of a suspect should change our belief in their guilt. The probability of guilt after the suspect has been identified, the posterior probability of guilt (PPG), can only be meaningfully estimated if we know the proportion of lineups that include a guilty suspect, P(guilty). Recent work used SDT to estimate P(guilty) on a single empirical data set that shared an important property with real-world data; that is, no information about the guilt or innocence of the suspects was provided. Here we test the ability of the SDT model to recover P(guilty) on a wide range of pre-existing empirical data from more than 10,000 identification decisions. We then use simulations of the SDT model to determine the conditions under which the model succeeds and, where applicable, why it fails.

**Results:**

For both empirical and simulated studies, the model was able to accurately estimate P(guilty) when the lineups were fair (the guilty and innocent suspects did not stand out) and identifications of both suspects and fillers occurred with a range of confidence levels. Simulations showed that the model can accurately recover P(guilty) given data that matches the model assumptions. The model failed to accurately estimate P(guilty) under conditions that violated its assumptions; for example, when the effective size of the lineup was reduced, either because the fillers were selected to be poor matches to the suspect or because the innocent suspect was more familiar than the guilty suspect. The model also underestimated P(guilty) when a weapon was shown.

**Conclusions:**

Depending on lineup quality, estimation of P(guilty) and, relatedly, PPG, from the SDT model can range from poor to excellent. These results highlight the need to carefully consider how the similarity relations between fillers and suspects influence identifications.

## Significance

Witnesses to crimes can provide crucial information to police investigators. Tragically, however, the identification of a suspect as the culprit can be simultaneously erroneous and compelling to the jury. Given that a witness has made an identification from a lineup procedure, how much weight should the court assign to that evidence? What we would like to know is the probability that the suspect is guilty, given that they have been identified by the witness. Although often ignored, a key step in answering this question is to determine the probability that the suspect is guilty *before the witness provides an identification*. This probability is the base rate of lineups that include the culprit, or guilty suspect, which can have a profound impact on the appropriate evaluation of an eyewitness identification in a courtroom. For an extreme example, in a police precinct in which the suspect is almost never guilty, even a suspect identification made with a high level of confidence should be treated skeptically. In a precinct in which the suspect is almost always guilty, however, even an uncertain suspect identification should be taken as a strong indication of guilt. Outside of a laboratory experiment, the true value of this base rate is unknown, of course. Here, we extend previous work and rigorously test a model-based statistical procedure for estimating this base rate. We find that the procedure works well when conditions matched those suggested as best practices for police lineups. This work paves the way to estimating the base rate of guilty suspects in real-world lineups.

## Introduction

Witnesses to crimes can provide crucial information to police investigators. Tragically, however, the identification of a suspect as the culprit can be simultaneously erroneous and compelling to the jury. Decades of research has focused on understanding how *estimator variables,* such as viewing distance, stress levels, and the presence or absence of a weapon, influence witnesses’ memory for the crime. Similarly, a substantial literature has explored the *system variables* that determine the details of the identification process, such as whether lineup members are viewed sequentially or simultaneously. Estimator and system variables work together to determine the potential accuracy of an eyewitness identification decision (e.g., Cutler, Penrod, & Martens, 1987; Wells, 1978).

Given that a witness has made an identification from a lineup procedure, how much weight should the court assign to that evidence? What we would like to know is the probability that the suspect is guilty, given that they have been identified by the witness. This value is called the positive predictive value (PPV) or the posterior probability of guilt (PPG, Wells & Lindsay, 1980; Wells, Yang, and Smalarz, 2015). PPG is defined as in Eq. 1

1 $$ PPG=P\left( guilty| identified\right)=\frac{P\left( identified| guilty\right)\times P(guilty)}{P\left( identified| guilty\right)\times P(guilty)+P\left( identified| not\ guilty\right)\times P\left( not\ guilty\right)}. $$

PPG is a specific form of a general equation known as Bayes’ Rule. The factors influencing PPG can be inferred from Eq. 1. The probability that the suspect is identified, if guilty, P(identified|guilty), is a function of both the estimator variables that determine the witness’s memory for the culprit and the system variables that render the identification decision easier or harder (e.g., biasing instructions and lineup administration method). As will become important below, if the suspect is innocent, physical similarity to the culprit will be among the factors influencing the probability that the suspect is erroneously identified, P(identified|not guilty) (Lindsay, 1986). These variables are relatively straightforward to interpret, if perhaps difficult to quantify in any given identification process. Equation 1 shows that the PPG increases under conditions that make the identification of guilty suspects more likely and the identification of innocent suspects less likely.

**Fig. 1:**
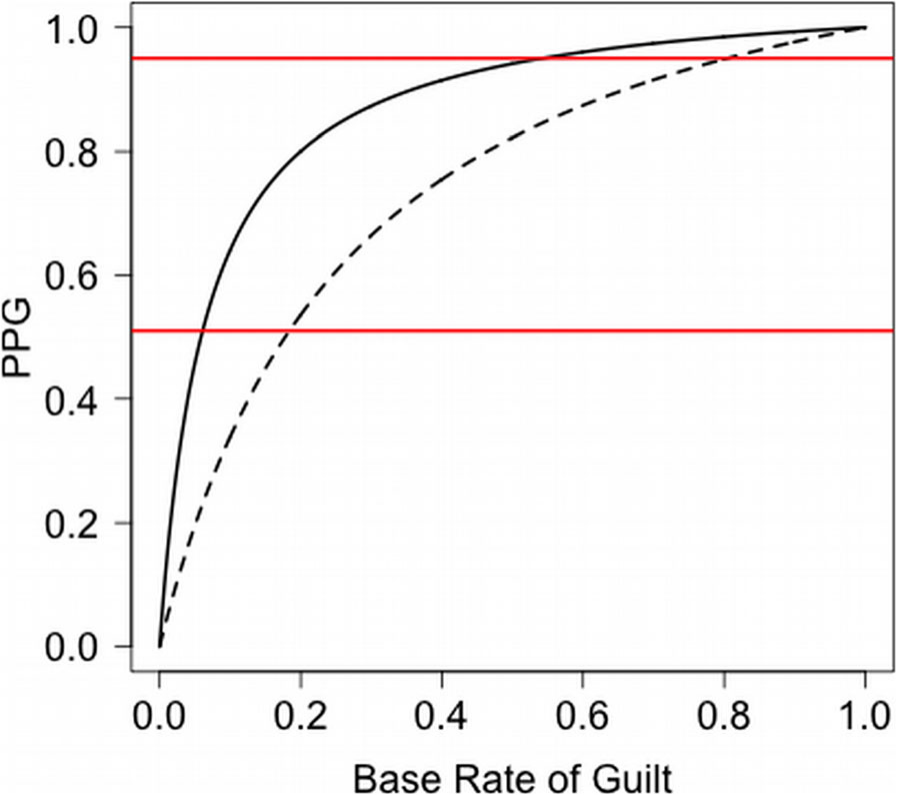
Posterior probability of guilt (PPG) as a function of base rate of guilt. Solid curve based on *d*’ = 1.8; dashed curve, *d*’ = 1.0 (*c* = 1 in both cases). Red lines at PPG = 0.95 (reasonable doubt) and 0.51 (probable cause)

A remaining component of Eq. 1, the prior probability that the suspect is guilty, P(guilty), reflects the base rate of lineups that include the culprit. Outside of a laboratory experiment, the true value of P(guilty) is unknown, and that fact presents a serious challenge to the utility of PPG because PPG varies dramatically as a function of base rate (Wells et al., 2015). Concretely, consider an example situation in which a witness’s memory is quite good and the system variables encourage the witness to respond conservatively, in accordance with the recommendations of the report of the National Research Council (2014), resulting in P(identified|guilty) = 0.46 and P(identified|not guilty) = 0.03. For this scenario, the solid curve in Fig. 1 shows PPG for every possible base rate, i.e., P(guilty). Note that PPG can range from 0 to 1, leaving it little more than a guess. If “reasonable doubt” is defined as a probability of guilt less than 0.95 (Simon, 1969), shown by the upper red line in Fig. 1, P(guilty) must be at least 55% to consider the suspect guilty. Under weaker identification conditions, P(guilty) must be even higher for that evidence threshold to be met. For example, the dashed curved in Fig. 1 shows that P(guilty) must be at least 81% when the witness with weaker memory is encouraged to respond conservatively, yielding P(identified|guilty) = 0.31 and P(identified|not guilty) = 0.07.

As discussed previously (see also, Wells, 1993), the prior probability or base rate of guilt, P(guilty), is unknown and may vary across law enforcement agencies and localities (Wells et al., 2015); indeed, 64% of law enforcement agencies have no written policy on the construction of a photo lineup (Police Executive Research Forum, 2013). Given this uncertainty, some researchers have argued for the use of a measure called *diagnosticity* (Wells & Lindsay, 1980). Diagnosticity is the likelihood ratio of identification of the culprit versus an innocent suspect:

2 $$ \frac{\kern0.50em P\left( identified| guilty\right)}{P\left( identified| not\ guilty\right)}. $$

The probability form of PPG in Eq. 1 can be written in odds form as follows,

3 $$ \frac{P\left( guilty| identified\right)}{\ P\left( not\ guilty| identified\right)}=\frac{P\left( identified| guilty\right)}{P\left( identified| not\ guilty\right)}\times \frac{P(guilty)}{P\left( not\ guilty\right)}. $$

Note that the diagnosticity ratio of Eq. 2 is included as the first term in the right-hand side of Eq. 3. This has led some researchers to argue that diagnosticity itself has probative value because larger likelihood ratios yield larger posterior odds of guilt regardless of the prior odds (Wells & Lindsay, 1980).

This use of diagnosticity, however, is problematic for several reasons. First, even a large value of diagnosticity does not necessarily imply high odds that the suspect is guilty – with low prior odds of guilt, P(guilty)/P(not guilty), the posterior odds, P(guilty|identified)/P(not guilty|identified), will also be low. For example, if diagnosticity is 15.3 (= 0.46/0.03), as for our example witness with stronger memory, but the prior odds of guilty are only 0.01, then the posterior odds of guilt are about 0.15, a result that strongly favors innocence. Second, diagnosticity is a performance measure that confounds memory strength and response bias, as well as other factors such as the number of photos in the lineup (Rotello & Chen, 2016). Thus, when available, PPG is a far better probative measure. Use of PPG, however, requires estimation of the base rate of lineups that contain the culprit to specify the prior odds of guilt.

Wixted, Mickes, Dunn, Clark, and Wells (2016) offered a substantial advance over the prior literature by providing a statistical tool that simultaneously allows estimation of witness-memory accuracy, response bias, and, crucially, the base rate of guilt. They fit a signal detection model (Macmillan & Creelman, 2005) to data for which the guilt or innocence of the suspect is not known, as is true in all identification procedures conducted by law enforcement. Using a novel analytic approach described below, Wixted et al. first demonstrated that the model accurately recovered a known base rate from empirical identification data even though the data fit by the model had no indication of which lineups had guilty and innocent suspects. As the authors noted, this feat is seemingly impossible, and one of our goals is to discover how the model is able to achieve it. Given this success, Wixted et al. then applied the same analysis to lineup identifications of robbery suspects in Houston, Texas (Wells, 2014), estimating that about 35% of those real lineups included a guilty suspect. This is a remarkable accomplishment. Wixted et al.’s method offers a tool capable of assessing and comparing the quality of investigative lineups across law enforcement agencies and locations. It also offers the possibility for PPG to be estimated more accurately, providing probative value to the courts.

In what follows, we first describe in detail how Wixted et al. (2016) inferred the base rate of guilty suspects from a single eyewitness identification experiment. We then extend and test the generality of the model’s success by using the same method to fit identification decisions collected across a wide variety of experiments with a total of 10,137 participant identifications, imitating the natural variations in lineup identifications conducted by law enforcement. In particular, we mixed data from a wide range of empirical studies including differences in simulated crime, lineup sizes, the similarity relations of fillers and suspects, exposure and retention intervals, the position of the suspect in the lineup, different types of biases, the presence of a weapon, the use of alcohol by the witness, and the particular individuals used in the lineups. Applying the model to this range of data is one of the main contributions of our work as it approximates the huge variability across real witnesses and lineup procedures better than any individual study that, by design, tightly controls estimator variables (see also, Lindsay, Read, & Sharma, 1998).

In follow-up analyses, we explored the conditions necessary for valid base-rate estimation using data from the 13 individual experiments of varying sample sizes and, in cases for which estimation was poor, we further evaluated illustrative conditions within those experiments. In each case, we used a bootstrapping process to overcome a design limitation inherent in the experimental work, namely that experiments typically present roughly half of their participants with lineups that include the culprit and half that do not. To test the model under a wider and more realistic range of possible base-rate conditions, we sampled identification decisions from a large data base such that the samples included 20%, 35%, 50%, 65%, or 80% guilty-suspect lineups (yielding 80%, 65%, 50%, 35%, or 20% innocent-suspect lineups); by repeating this process many times, we were able to assess the variability across outcomes. We then performed parameter-recovery simulations with data generated by the model to determine the theoretical limits on the ability of the model to accurately estimate base rates, and to explore *how* the model can provide information about the proportion of guilty suspects without knowledge of whether or not any of the individual suspects were guilty or innocent.

To preview our results, we found that, in many cases, the model successfully recovered the simulated base rate and other model parameters reasonably well, but some systematic and informative recovery errors were observed. We show that these errors occur in situations where the model can be plausibly understood to be misspecified. In particular, experiments with various forms of bias were problematic. Finally, we consider the implications of these findings and return to estimation of the PPG that would be highly valuable to the courts.

### The signal detection theory (SDT) model

**Fig. 2:**
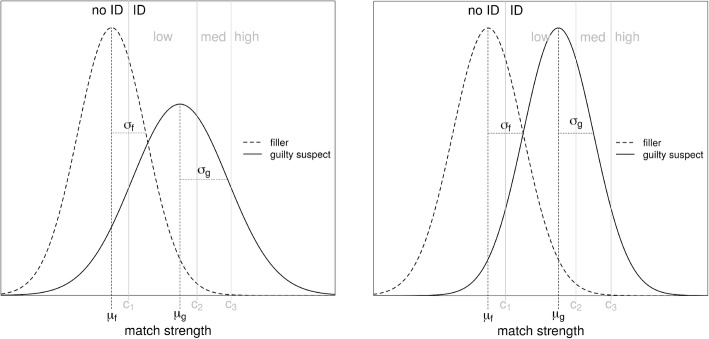
The signal detection model for lineups. Left: unequal-variance model. Right: equal-variance model. *μ*_g_ and *σ*_*g*_ are the mean and standard deviation of the guilty-suspect distribution. *μ*_f_ and *σ*_*f*_ are the mean and standard deviation of the filler distribution. *c*_1_, *c*_2_, *c*_3_ are the response criteria for the low-, medium- (med), and high-response confidence regions. ID and No ID are response regions in which an identification is or is not made, respectively

In a typical six-person lineup, there is one suspect and five fillers (also called “foils” or “lures”) who are known to be innocent (e.g., Wells & Turtle, 1986). In what follows, we assume a simultaneous lineup, that is, all six individuals are viewed at the same time. If the suspect is guilty, we assume that the witness’s memory will be stronger for that individual than for any of the fillers, at least on average (*μ*_g_ – the subscript *g* stands for “guilty”). Because the fillers are selected to share many physical characteristics with the suspect, such as age, race, and hair color, they will also have some strength in memory (with mean *μ*_f_ – the subscript *f* stands for “filler”). In fact, it is possible that one or more fillers will seem more familiar to the witness than the suspect. This can occur when the suspect is innocent, of course, but it can also occur by chance when the suspect is guilty. This variability in memory strength is modeled with Gaussian distributions, *N*(*μ*_g_, *σ*_*g*_) and *N*(*μ*_*f*_, *σ*_*f*_), as shown in the left panel of Fig. 2.

Witnesses are assumed to make identification decisions by comparing the highest memory strength elicited by a lineup photo to SDT-model response criteria (e.g., *c*_1_, *c*_2_, *c*_3_); a memory strength that exceeds *c*_1_ but not *c*_2_ results in positive identifications with low confidence, those that exceed *c*_2_ but not *c*_3_ yield identifications with medium confidence, and those that exceed *c*_3_ are high-confidence decisions. If none of the faces in the lineup elicit a memory strength that exceeds *c*_*1*_ then the lineup is rejected with a “no ID,” “reject,” or “not here” response. With three confidence levels, the data have 12 degrees of freedom (*df*) total, six each from the two types of lineup (guilty or innocent suspect). Each lineup type has six *df* because there are seven possible responses (identify the suspect with one of three confidence levels, identify a filler with one of three confidence levels, or reject the lineup) with one *df* lost because the seven response frequencies must sum to the total number of identification attempts. The model has five free parameters (*μ*_g,_*σ*_*g*_, *c*_1_, *c*_2_, *c*_3_; without loss of generality, *μ*_f_ = 0 and *σ*_*f*_ = 1). This model has been shown to work well when we know the guilt or innocence of the suspect, as is always true for experimental designs (Mickes, Flowe, & Wixted, 2012; for a summary, see Gronlund & Benjamin, 2018).

Estimating the base rate of guilty suspects involves fitting identification data for which the guilt or innocence of the suspect in the lineup is not known, as in all investigative lineups. Wixted et al. (2016) called this type of data *collapsed*. The data now only have six degrees of freedom because the lineups are collapsed into the same set of frequencies for the seven available responses (suspect or filler identifications at low, medium, or high confidence or a lineup rejection). The model must now include a parameter to estimate the base rate of guilty suspects, *p*_*g*_, where the subscript *g* again stands for “guilty.” To gain a degree of freedom in the model, Wixted et al. fixed the standard deviation of the target distribution, *σ*_*g*_ = 1, thus assuming an equal-variance model as shown in the right panel of Fig. 2. Although this equal-variance assumption does not typically hold in a standard recognition task (e.g., Ratcliff, Sheu, & Gronlund, 1992), it might hold for a lineup task where there is a single target (the guilty suspect) but many potential foils, inflating the relative variability of the lure distribution (Wixted et al., 2016; Wixted, Vul, Mickes, & Wilson, 2018).

It is interesting to note that the recovery of base-rate information using a SDT model is not ordinarily possible; for example, collapsing confidence-rating data from a standard old/new recognition experiment would make it impossible to estimate memory parameters or determine the proportion of target trials. Generally, it seems incredible to claim that one could measure the *proportion* of guilty suspects without knowing whether any one of the individual suspects is guilty or innocent. As will be explained in more detail below, it is the unique features of a lineup, in particular, the inclusion of fillers who are known to be innocent, that allows the model to accomplish this seemingly magical feat.

### Wixted et al. (2016) model fits

Wixted et al. (2016) fit an equal-variance version of the model to data previously published in Palmer, Brewer, Weber, and Nagesh (2013). The model simultaneously predicted the probability of suspect and filler identifications at each confidence level. Indeed, the mixtures of identifications across confidence levels are essential to the ability of the model to estimate the base rate of guilt, as we show in model simulations below. Wixted et al. also sampled guilty- and innocent-suspect trials from the Palmer et al. data set in varying proportions, concluding that the SDT model accurately recovered the sampled base rate over most of the range, though the base-rate parameter *p*_*g*_ underestimated the true value for base rates over 0.80. Using the same analytic approach, Wixted et al. fit the SDT model to the Houston robberies field data, estimating *p*_*g*_ to be 0.35. Although there is no way to verify the accuracy of that estimate, it is worth noting that this work is the first principled estimate of a real-world eyewitness base rate and is based on the same signal detection model that has recently led to other advances in the field (e.g., Colloff & Wixted, 2020; Semmler, Dunn, Mickes, & Wixted, 2018; Wixted et al., 2018). Furthermore, as we show below, the model used to make this estimate works well under standard testing conditions, indicating that this estimate should be taken seriously.

The advance offered by Wixted et al. should not be underestimated. Consider the implications for the courts: If the estimates of the base rate of guilty suspects can be trusted, this model would allow far more accurate estimation of the posterior probability that an identified suspect is guilty. For example, reconsider our stronger and weaker witnesses discussed in the context of Fig. 1. Without an estimate of *p*_*g*_ we were left with estimates of PPG that ranged from 0 to 1 for both witnesses. If they were witnesses to a robbery in Houston, we can refine our estimates of the PPG for the suspect to roughly 0.9 for the stronger witness, and about 0.7 for the weaker witness, assuming that the base rate of lineups containing guilty suspects is 0.35 for that type of crime in that location.

Before reaching a conclusion with such strong and consequential implications, however, it is necessary to test the signal detection model on a wider range of data. The current work fills this gap. To preview our results, the model generally works well, though not always as well as for the Palmer et al. data fit by Wixted et al., and the situations in which the model is challenged by the data prove to be informative about what type of data cause the model to be misspecified, thereby producing poor parameter estimates.

## Results

Details of the experimental data, SDT model and model-fitting procedure, and the simulations are provided in the “Methods” section. Here we focus on the results and only briefly describe the methods. We do note, however, that, rather than relying on simulations, closed-form solutions were used for the SDT model (e.g., Cohen et al (n.d.) (in press)).

### Experimental data

#### Full data set

The SDT model described previously was fit simultaneously to the data from 13 eyewitness lineup experiments, involving a total of 10,137 identification decisions. As discussed previously, the rationale for mixing data across a large number of studies was to approximate the huge variability across real witnesses, who view different crimes under different viewing conditions and who vary substantially in their individual characteristics. We do not claim that our combined data set is a close analog to real witness identification data, but it is certainly a much closer analog than data from any single experiment in which these estimator variables are all tightly controlled (or manipulated) across subject-witnesses. All of the 13 experiments collected confidence ratings from witnesses and used a simultaneous lineup procedure, i.e., all photos were shown at the same time. Critically, the model was applied to collapsed data, i.e., data in which the guilt or innocence of the suspect is not known. Thus, the main question is whether, without this information, the model can recover the base rate of guilty suspects present in the data by estimating a value for the *p*_*g*_ parameter that is near the true P(guilty).

**Fig. 3:**
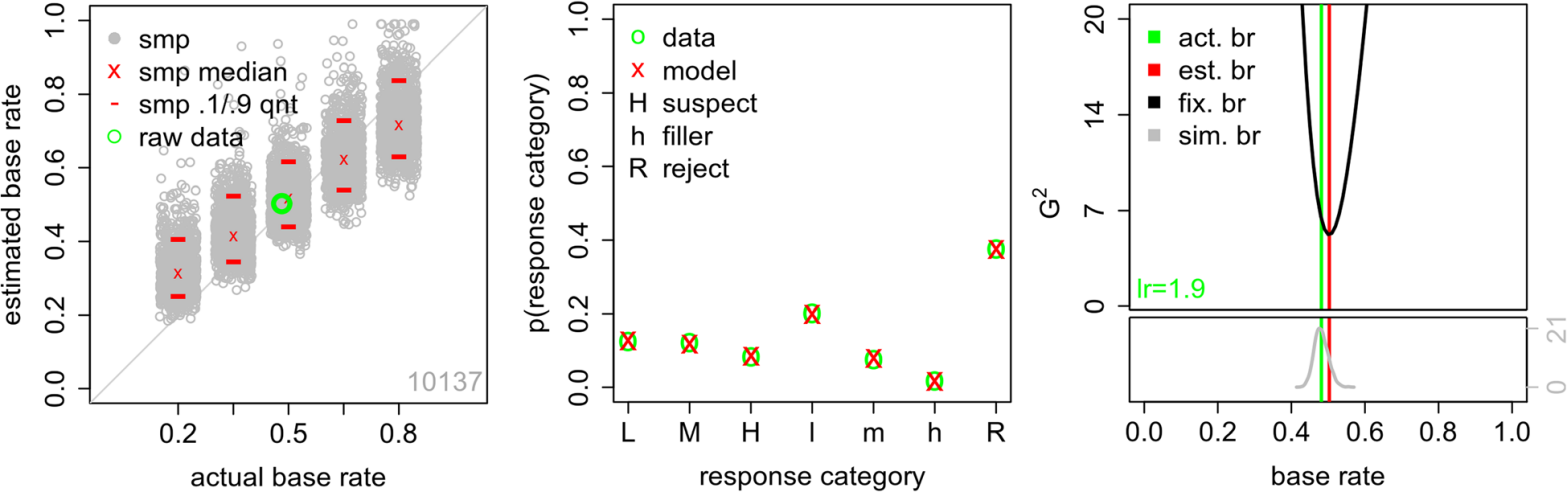
Results of the signal detection theory (SDT) model analysis when fit to the full data set. Left: actual and estimated base rates for the full data set combined across all 13 experiments. The green circle shows the actual experimental base rate and the SDT-model estimate. Each gray circle shows the estimated base rate for one of the sampled (smp) data sets, with the sampled base-rate value jittered for visibility. The red lines and red x show the 10th and 90th quantiles and median of these estimated base rates. The number in the lower-right corner is the overall sample size. Middle: data results and model predictions for low- (L), medium- (M), and high- (H) confidence suspect identifications, low- (l), medium- (m), and high- (h) confidence filler identifications, and lineup rejections (R). Right: the green line shows the actual base rate in the experimental data and the red line shows the estimated base rate from the SDT model. In the upper section, the black curve shows how the model fit value changes as the model base rate varied. The number in the lower-left provides the likelihood ratio (lr) of the model when the estimated and actual experimental base rates are used. In the lower section, the gray curve shows the distribution of estimated base rates for data simulated from the SDT model with the model base rate fixed at the actual experimental base rate. The *y*-axis is frequency

The results are shown in Fig. 3. First, consider the left panel. The number in the lower right shows the sample size in the original data set. The green circle plots the estimated base rate for the experimental data on the *y*-axis against the actual experimental base rate on the *x*-axis. The model does an excellent recovering the actual experimental base rate.

Next, we bootstrapped samples from the original data set to generate data sets with a known range of base rates. That is, samples of trials in which the suspect was known to be guilty were combined with samples of trials in which the suspect was known to be innocent in different proportions, i.e., .20, .35, .50, .65, and .80 guilty suspects. Each sampled data set comprised 1000 identification attempts, so, for example, a .20 base-rate data set would have responses from 200 guilty-suspect lineups and 800 innocent-suspect lineups. We refer to data generated in this way as *sampled data* and the base rates of sampled data as *sampled base rates*. The SDT model was then used to estimate the sampled base rate. This process was repeated 1000 times. The gray circles represent the estimated sampled base rate for each of the 1000 sampled data sets, with the sampled base rates jittered on the *x*-axis for visibility. For each sampled base rate, the median estimated base rate is marked with a red x and the 10th and 90th quantiles are marked with red lines.

Consistent with Wixted et al. (2016), the estimated base rate tracks the actual sampled base rate well. There are some caveats, however. First, there is considerable variability in the estimated base rate for each actual sampled base rate. The middle 80% of the estimated base rates, i.e., the distance between the 10th and 90th quantiles, span a range of approximately 0.15 to 0.20. Although this result indicates that the model’s estimated base rate provides a fair approximation to the true value, it is an open question as to whether the observed level of variability in those estimates is acceptable. This variability will also change with sample size, and readers should note that the displayed estimates are based on a large sample (1000 witnesses). Second, there is a slight but clear bias such that low sampled base rates tend to be overestimated. To a lesser extent, high sampled base rates tend to be underestimated. Base-rate estimation is best for sampled values near 0.5. While we might be tempted to take comfort from the fact that most experimental base rates are also near 0.5, there is typically no need to estimate experimental base rates; rather, our goal is to accurately estimate the currently unknown base rate of police line ups that include the guilty suspect, which may vary considerably.

The middle panel of Fig. 3 shows the fit of the model to the original data. The green o’s are the experimental data and red x’s are the model predictions. The labels L, M, and H show the proportion of trials resulting in low-, medium-, and high-confidence suspect identifications. Similarly, the labels l, m, and h reflect the proportion of trials yielding low-, medium-, and high-confidence filler identifications, and the point labeled R shows the proportion of lineups that were rejected by the witness (i.e., a “no ID” decision was made). The fit is excellent, with the model accurately predicting performance in every response category.

The bootstrapped samples shown in the left panel of Fig. 3 provide one way of evaluating how well the SDT model estimates the sampled base rate. A different way of assessing the model is to consider how well it fits the data when a particular base rate is assumed. Concretely, suppose that the true base rate in a particular data set is 0.50. If the SDT model provides a good description of the data, it should provide a good fit (indicated by a small value of G^2^) when the base-rate parameter *p*_*g*_ is fixed at a value close to 0.50, and a relatively poor fit (i.e., large G^2^) when *p*_*g*_ is set to a value that is far from 0.50. Observing such a pattern would provide support for the conclusion that the model’s estimated base rate is constrained by the data. On the other hand, if changes in the model’s base-rate parameter do not result in substantial changes in the fit statistic, then that would imply that the model’s estimated base rate is not sufficiently informed by the data and should not be trusted.

The right panel of Fig. 3 shows the results of this sensitivity analysis. The upper section of the right panel shows the fit measure, G^2^, that is observed when the SDT model is fit to the experimental data under a wide range of assumptions about the value of *p*_*g*_. Specifically, we fit the experimental data many times, using a different fixed value of *p*_*g*_ (ranging from 0.01 to 0.99) for each fit. The *x*-axis shows the fixed value of the model base rate *p*_*g*_ and the black curve shows the corresponding values of G^2^ (where lower values are better). For this large data set, with a true base rate of approximately 0.52, the model’s fit profile is quite good. The parabolic shape of the black curve shows that the model only fits these data well when the model’s base-rate parameter is in a relatively narrow range around the true base rate in the data (shown with the green vertical line). This parabolic shape shows that it is not a coincidence that the model estimates the base rate in the data quite accurately when the base-rate parameter is free to vary (as in the results shown in the left panel of Fig. 3). This estimated value is shown with the red vertical line.

Another advantage of this approach is that we can compare the fit of the model when the true base rate is used as the value of *p*_*g*_ to the fit that results when *p*_*g*_ is unconstrained. That is, we can compute the relative likelihood, *lr*, of the model with the best-fitting estimated base rate and the actual base rate. This value, shown in green in the lower-left corner of the right, upper panel, means that the best-fitting base rate is 1.9 times more likely than the actual base rate. In this context, that means that fixing the model base rate to the actual base rate does not meaningfully change the fit of the model.

Finally, the lower section of the right panel of Fig. 3 assesses how well the SDT model can estimate base rates when the data are known to be generated by the model, assuring that the model assumptions are met. This section of the figure was generated as follows. First, the model was fit to the full data set with *p*_*g*_ fixed at the actual data base rate. Second, data were simulated from the model using the parameters estimated from the first step and *p*_*g*_ was again fixed at the actual base rate. The sample size and lineup sizes for the simulation were the same as in the data. Third, the model was fit to this simulated data. Steps 2 and 3 were repeated 1000 times. The lower section of the right panel of Fig. 3 provides the frequency with which a given base rate, *p*_*g*_, was recovered. The gray curve shows a kernel density estimation of the distribution of estimated base rates. The peak of the distribution is at the generating base rate, provided by the green vertical line. Critically, the estimated base rate from the full data set, the red line, is still relatively likely.

In summary, all of these different ways of evaluating the ability of the SDT model to fit these data suggest that it does a good job, although there is quite a bit of variability in the estimated base rate even with a sample that includes many identification attempts (*N* = 1000 trials, left panel of Fig. 3).

**Fig. 4:**
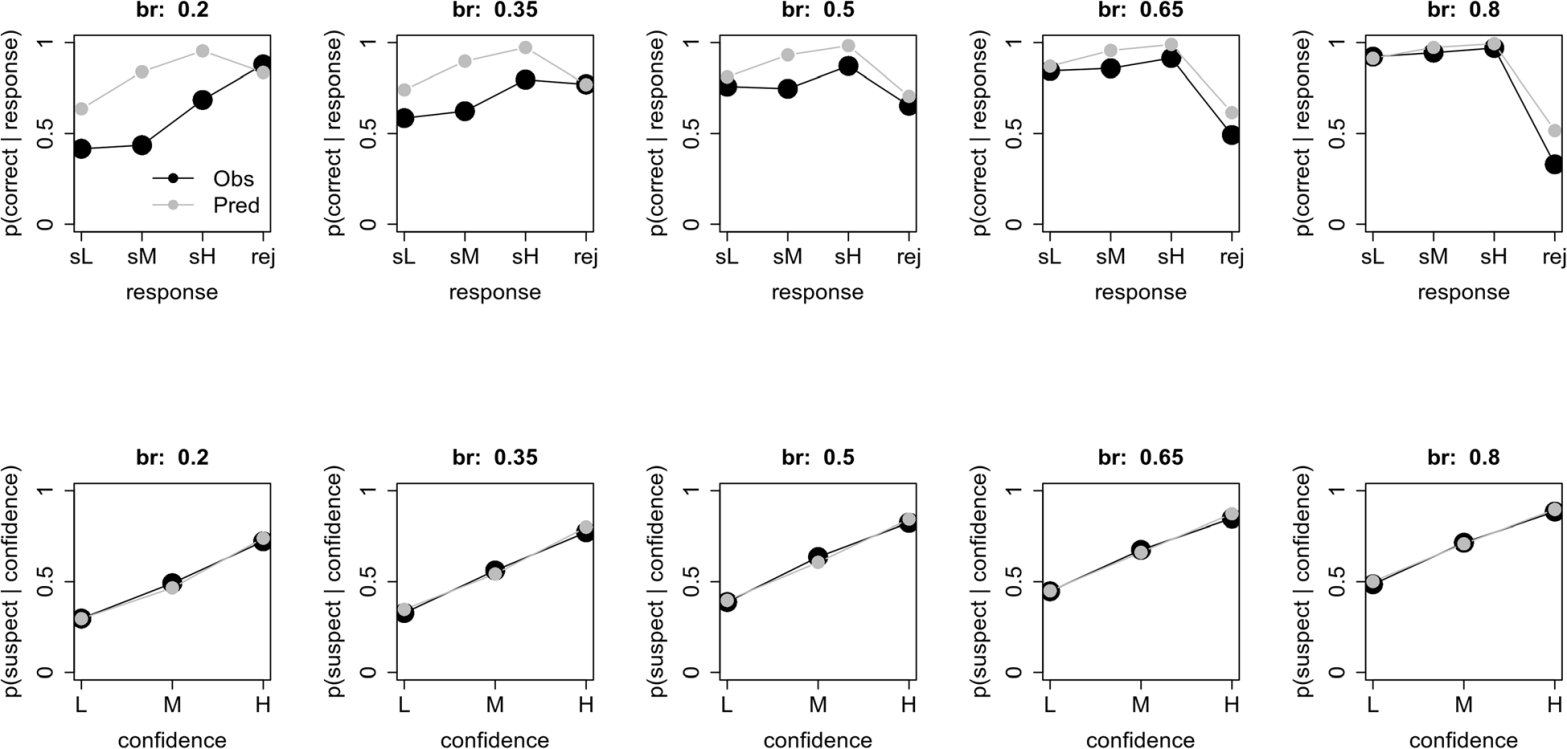
Calibration curves using the full data set for both experimental data and model predictions. Each column is for a different sampled base rate. The top row plots the proportion of correct responses given either a rejection (rej) or a low (sL)-, medium (sM)-, or high (sH)-confidence suspect response. The bottom row plots the probability of a suspect identification given a low- (L), medium- (M), or high- (H) confidence response (suspect or filler)

A more familiar way of looking at eyewitness identification decisions is to use a calibration curve (Juslin, Olsson, & Winman, 1996) or confidence-accuracy characteristic (Mickes, 2015), which plots the accuracy of the identifications as a function of witness confidence. These curves can be generated for both empirical data and model predictions, as shown in Fig. 4. Each column shows the calibration curves for a sampled base rate. The model predictions are averaged across the results from all simulations at that base rate. We begin by discussing the top row, which shows the proportion of correct responses given either a rejection (rej) or a low (sL), medium (sM), or high (sH) confidence suspect identification. For example, a value of 0.60 for sL means that 60% of the low-confidence suspect choices were to guilty suspects and 40% were to innocent suspects. A value of 0.70 for rej means that 70% of the rejected lineups included an innocent suspect, and so were correctly rejected; the other 30% included a guilty suspect that was missed. These results illustrate an effect of the over-prediction of estimated base rate for low-sampled base rates (left panel of Fig. 3). Specifically, for low-sampled base rates, the model predicts that performance for suspect identifications will be more accurate than is actually observed. As sampled base rate increases, this difference between the data and the model’s predictions is reduced; however, the model slightly over-predicts the accuracy of rejected lineups.

**Fig. 5:**
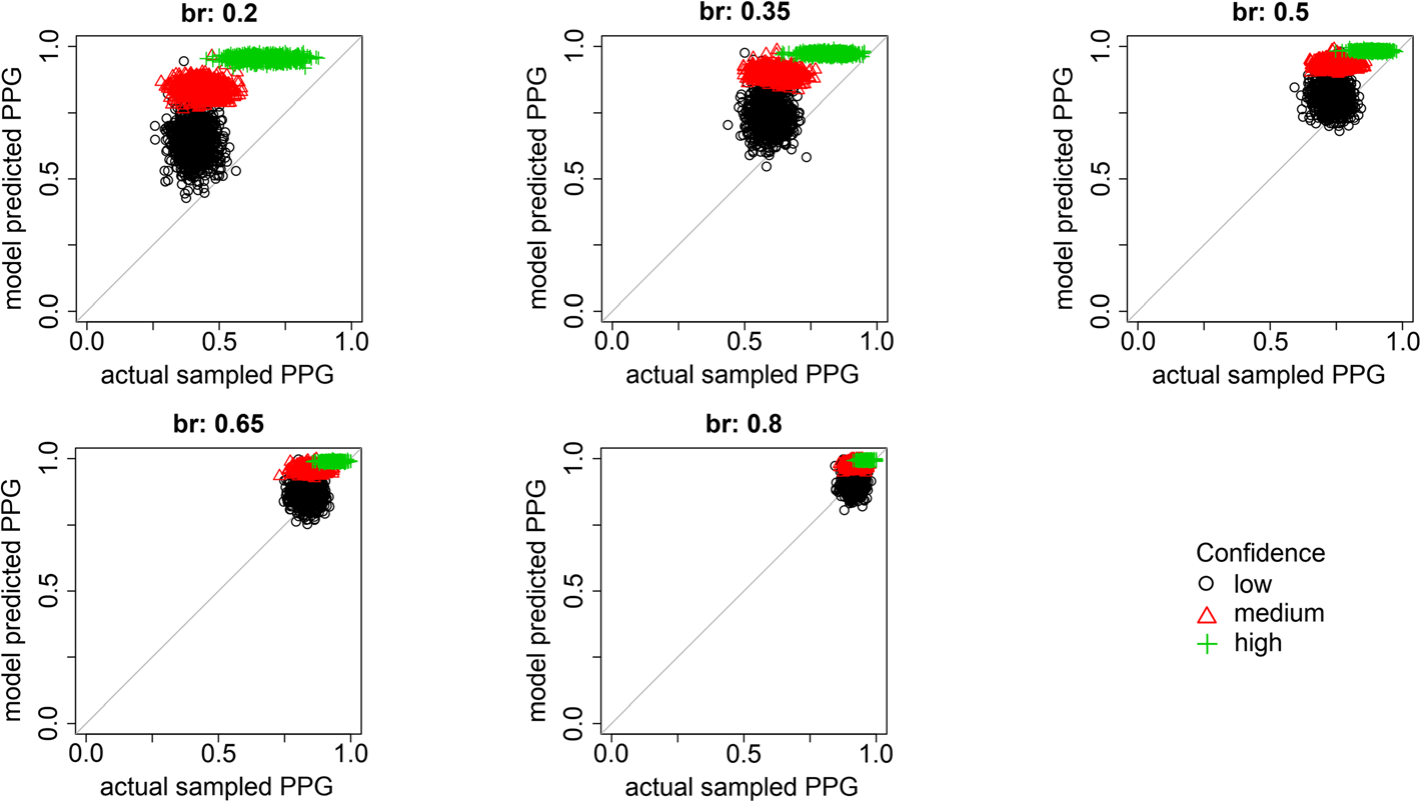
The model-predicted posterior probability of guilty (PPG) plotted against the actual sampled PPG for different base rates sampled from the full data set, for low-, medium-, and high-confidence responses

These graphs also reflect the ability of the SDT model to recover PPG. Recall that PPG is defined as the probability of the suspect being guilty, if the suspect is identified. That is exactly the information the sL, sM, and sH points in the top row of Fig. 4 provide, for individual confidence levels. For comparison to previous work, those graphs were generated by averaging across predictions. To get a sense of the variability inherent in the estimated PPG we repeated this analysis, but for each individual sampled data set. The results are provided in Fig. 5. There are three main results. First, the model captures the relative PPG values across both base rates (panels) and low (black circles), medium (red triangles), and high (green crosses) confidence levels. Second, however, and consistent with Fig. 4, the model over-predicts PPG, especially for low base rates. Third, there is considerable variability across sampled data sets, again, especially for low base rates, suggesting that estimated PPG is a relatively imprecise measure of actual PPG. This result is important as it suggests that, although estimation of base rates is fairly good, the PPG estimated by the model is significantly higher than the PPG in the data, especially for low base rates similar to that estimated by Wixted et al. (2016) for the Houston data. That is, the base rate estimated from the model provides an inflated sense of the guilt of an identified suspect.

Returning to Fig. 4, the bottom row plots the probability of a suspect identification given a low- (L), medium- (M), or high- (H) confidence response (for either a suspect or filler identification). For example, a value of 0.30 for low-confidence responses means that 30% of the low-confidence responses were suspect identifications and 70% were not. Here, the model does an excellent job throughout.

#### Individual experiments

The previous results show that, when applied to a data set drawn from a range of experimental procedures, sample sizes, lineup sizes, and manipulations that simulate different estimator variables, the model does a fairly good job of estimating the actual base rate, but with systematic biases in some cases and high variability in estimates. To get a sense of how robust the results were, we next applied the SDT model to the data from each of the 13 individual experiments, repeating the same analyses on each experiment as we reported for the overall data set. The only difference is that the analyses were applied to each of the 13 experiments individually, rather than the combined data set.

Overall, these individual experiment model fits were excellent. That is, the SDT model did an excellent job in accounting for the response proportions for each identification category. These data, along with the calibration curves and sensitivity analyses for each experiment, are provided on Open Science Framework (OSF;  https://osf.io/3qz5n).

**Fig. 6:**
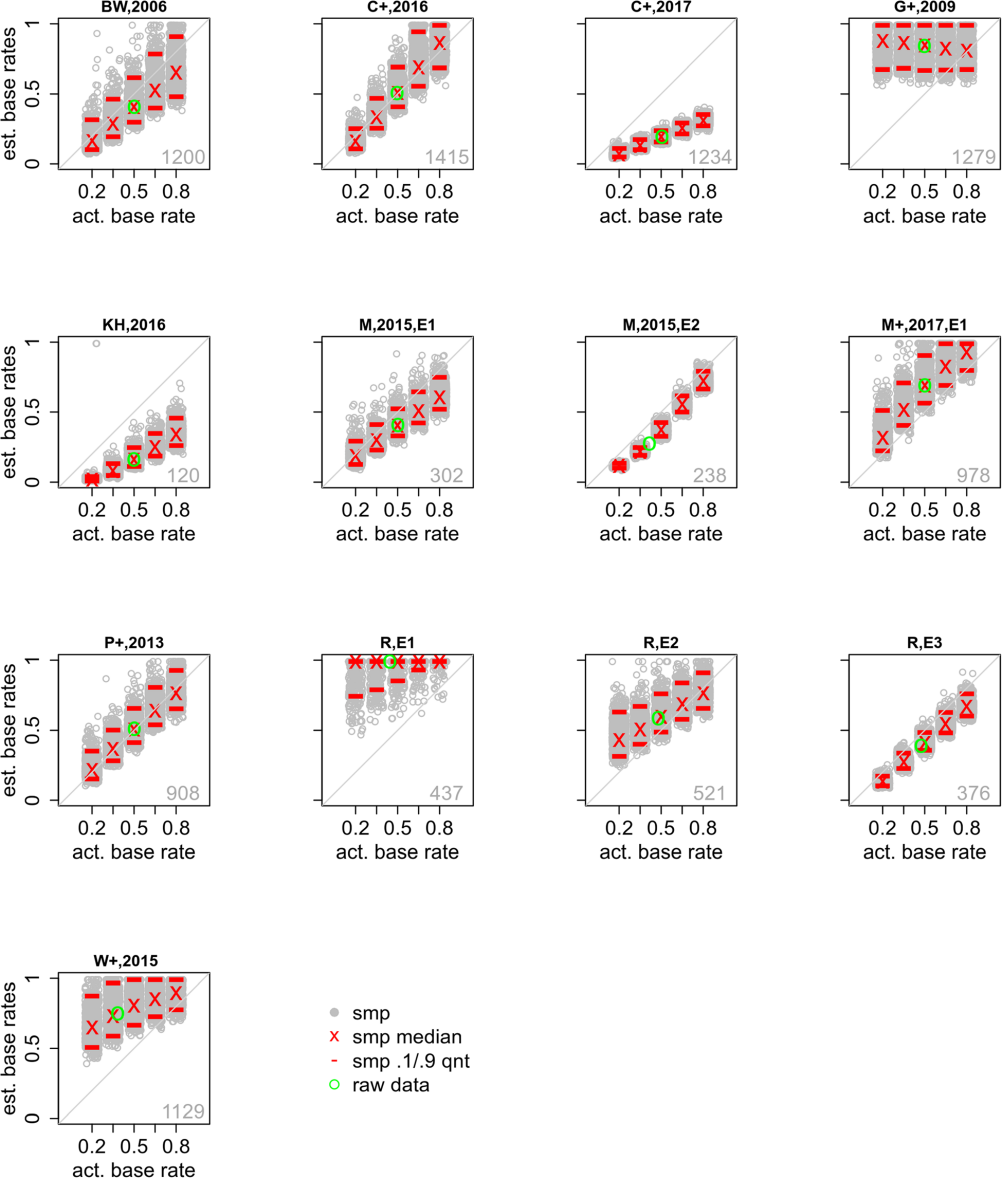
Actual and estimated base rates for each of the 13 individual experiments (see Table 1). The green circle shows the actual experimental base rate plotted against its estimate. Each gray circle shows the estimated base rate for one of the sampled data sets (smp; the sampled base rates are jittered for visibility). The red lines and red x show the 10th and 90th quantiles and median of these estimated base rates. The number in the lower-right corner is the experiment sample size

The key results of these model fits are the base-rate estimates for the bootstrapped samples drawn for each experiment; these are shown in Fig. 6. The results are quite variable. For some experiments, the model did a very good job in estimating the base rate. For example, the data from Brewer and Wells (2006), Carlson et al. (2016), Mickes (2015) Experiments 1 and 2, and Rotello et al. (2015) Experiment 3, produced very good to excellent mean estimated base rates (though often with high variability). Of particular note is the superb base-rate estimation for the Palmer et al. (2013) study which is the same data set used by Wixed et al. (2016). The base-rate estimates for other studies were good, albeit biased to varying degrees, including Mickes et al. (2017) Experiment 1 and Rotello et al. (2015) Experiment 2. Yet other studies showed a good correlation between actual and estimated base rates, but with an extreme bias. These include Carlson et al. (2017), Kneller and Harvey (2016), and Wetmore et al. (2015). The estimated base rates for Rotello et al. (2015) Experiment 1 and Gronlund et al. (2009) were very poor.

Mediocre to poor base-rate estimation in some of these studies can be easily explained. In Rotello et al. Experiment 1, the suspect identification rates were quite low and did not vary much as a function of confidence. The Kneller and Harvey (2016) study involved only 120 participants, and in two of their three conditions there were no suspect identifications made with high confidence. These results suggest that it is important to get a good sample of suspect identifications and responses at all confidence levels. The Wetmore et al. (2015) study, like Gronlund et al. (2009), included biased lineups that strongly encouraged selection of the suspect, which results in an inflated estimate of the base rate of guilty suspects. We suspect that this is one main reason the that model overestimates the base rate for this experiment and we explore this explanation further in the next section.

#### Experimental conditions

Estimation of base rates for the individual experiment data shows highly variable performance. For the majority of studies, base-rate estimation was good to excellent. There were studies, however, for which base-rate estimation was very poor. In some cases, as discussed previously, an explanation is readily available. In others, it is not readily apparent why the model performs so poorly. To generate a more fine-grained view of where the model performs well and where it performs poorly, we now turn to a condition-by-condition analysis of the data.

A summary of the conditions used is provided in Table 2 of the “Methods” section. The full set of results, the model fits (which were all very good to excellent), sensitivity analysis, and calibration curves for each condition are provided on OSF. Here, we discuss the conditions from two experiments that proved to be especially informative regarding the conditions under which the model performed poorly.

Some of the experimental manipulations are expected to strongly influence identification rates and the distribution of confidence. For example, biased or unfair lineups in which the fillers are dissimilar to the suspects, as in Gronlund et al. (2009), tend to inflate suspect identification rates and confidence levels. The presence of a visible weapon, as in Carlson et al. (2017), is expected to draw attention away from the perpetrator, thus reducing guilty-suspect identification rates and lowering confidence. Indeed, estimation of base rate was especially poor in exactly these conditions.

**Fig. 7:**
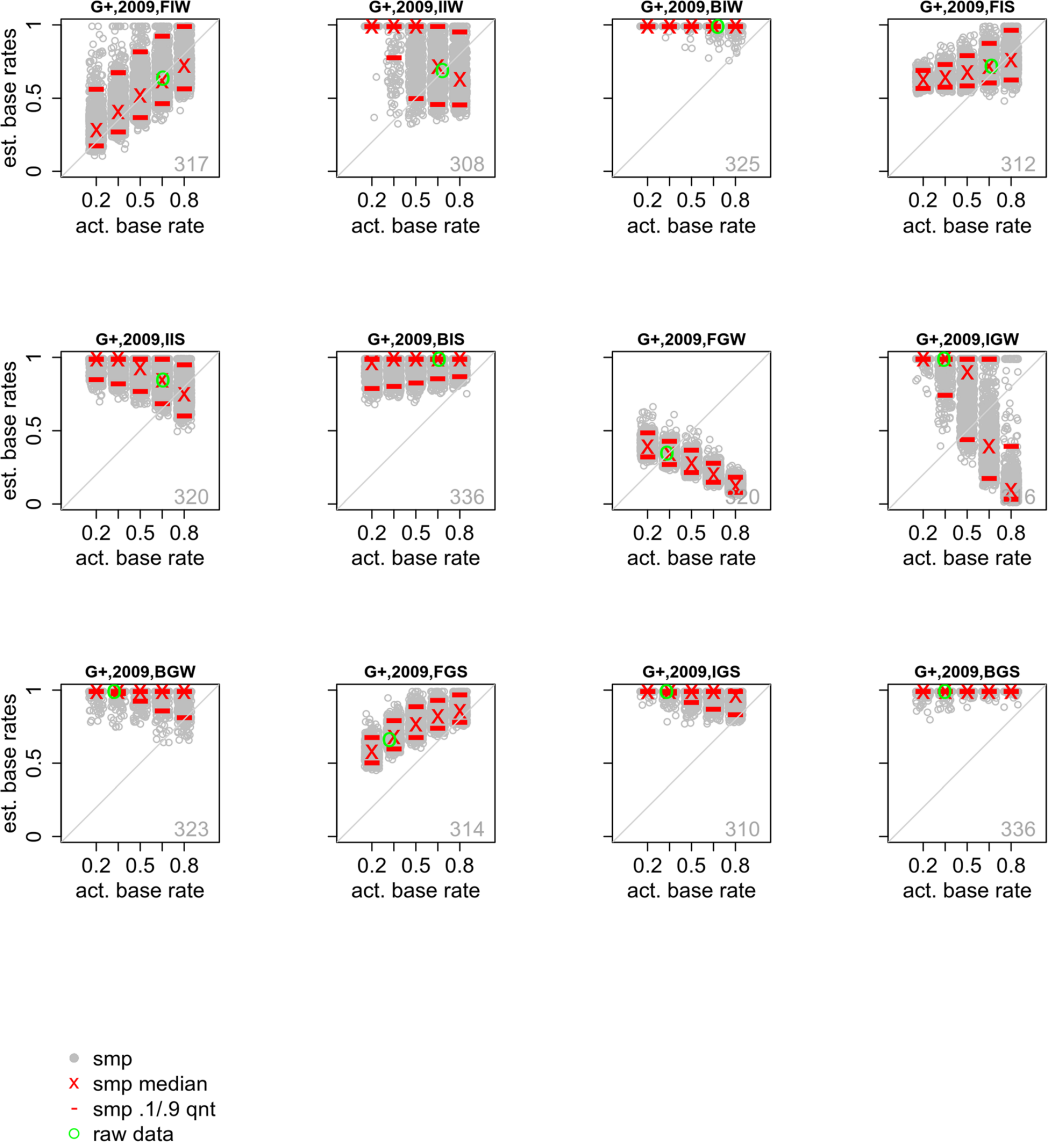
Actual and estimated base rates for conditions from Gronlund et al. (2009). The green circle shows the actual experimental base rate and its estimate. Each gray circle shows the estimated base rate for one of the sampled data sets (smp). The red lines and red x show the 10th and 90th quantiles and median of these estimated base rates. The number in the lower-right corner is the condition sample size. See Table 2 for condition labels

The Gronlund et al. (2009) study manipulated a number of factors, including the fairness of the lineup and the memory strength of the suspect. There were three levels of fairness, fair (F), intermediate (I), or biased (B), which were generated by manipulating the similarity of the fillers and suspect. In addition, guilty suspects could be represented by a photo that was a better or worse match to the way that they appeared in the witnessed crime, resulting in a strong (GS) or weak (GW) memory strength. Naturally, the GS conditions resulted in more suspect identifications than the GW conditions. Similarly, innocent suspects could be strong (IS) by virtue of offering a good match to the perpetrator, or else weak (IW); there were more IS than IW suspect identifications. Interestingly, the Gronlund et al. data reveal that the GW suspects were identified less often than either the IS or IW suspects; in that case, the perpetrator elicited lower memory strength than the innocent suspect. Because the innocent suspects had never been seen before, these conditions could be viewed as manipulations of selection bias rather than memory strength. The condition-by-condition Gronlund et al. (2009) results are provided in Fig. 7.

The Gronlund et al. (2009) results are nuanced. Estimation was good, albeit noisy, for the fair (no-bias) condition with a weak innocent suspect (FIW). This condition corresponds to a standard lineup in which the weak innocent suspect was essentially just another filler. All of the other conditions, however, show strong deviations from accurate base-rate estimation. Base-rate estimation is at or near ceiling for all of the biased (B) conditions because in those conditions the suspect was identified with high probability and high confidence, regardless of guilt or innocence; the model interprets this response pattern as reflecting a high base rate of lineups containing guilty suspects. One way of understanding this outcome is to contrast the *effective* size of the lineup (E’; Tredoux, 1998) and the lineup size assumed by the model, which always reflects the actual number of photos shown. To the extent that these two values differ, the model is misspecified for the data. Whereas the model assumed a lineup size of six, for the biased lineups from Gronlund et al. (2009), the effective lineup size E’ was always less than two.

Another striking misestimation of the sampled base rate is evident in instances of the Gronlund et al. (2009) data in which the guilty suspect is a poor match to the perpetrator (GW). In that case, the model’s estimates of base rate are actually negatively correlated with the sampled base rate. This failure of the model occurs because the GW suspect is selected less often than either the IW or IS suspects, which means that there are fewer suspect identifications in the data as the sampled base rate increases; the model interprets this low suspect identification rate as reflective of the base rate. The combined effect of (intermediate) biased lineups and guilty suspects that are a poor match to the perpetrator is visible in the IGW condition, which shows overestimation of the base rate overall due to the relative dissimilarity of the filler photos to the perpetrator, as well as the negative relationship between estimated and sampled base rates that stems from inclusion of the GW suspect.

**Fig. 8:**
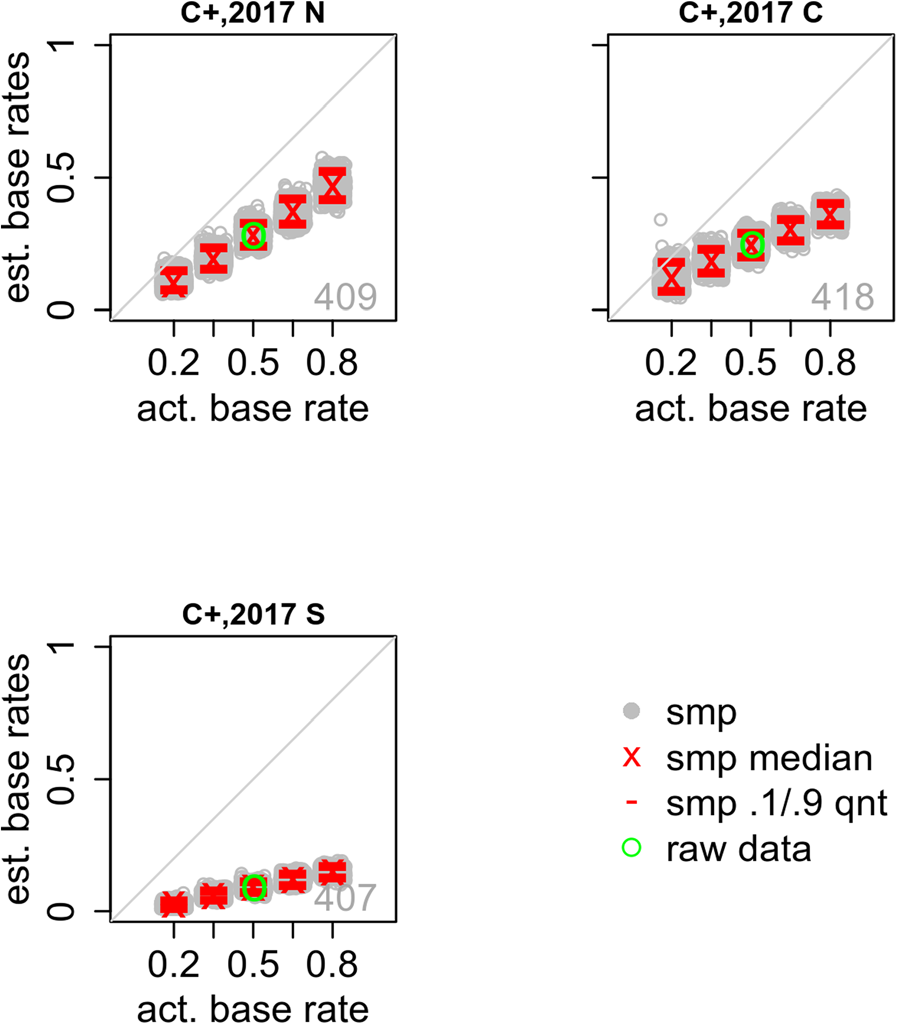
Actual and estimated base rates for conditions from Carlson et al. (2017). The green circle shows the actual experimental base rate and its estimate. Each gray circle shows the estimated base rate for one of the sampled data sets (smp). The red lines and red x show the 10th and 90th quantiles and median of these estimated base rates. The number in the lower-right corner is the condition sample size. See Table 2 for condition labels

In Carlson et al. (2017) a weapon was either shown (S), present but concealed (C), or not shown (N). The condition-by-condition Carlson et al. (2017) results are provided in Fig. 8. In all conditions, the model’s estimate of the base rate is too low, and the degree of underestimation varies systematically with the participants’ awareness of the weapon. When there is no weapon (N), the bias is smallest. When the weapon is visible (S), the estimates are very strongly biased, reflecting the relatively low probability that the suspect is identified. Presence of a concealed weapon results in moderate bias.

**Fig. 9:**
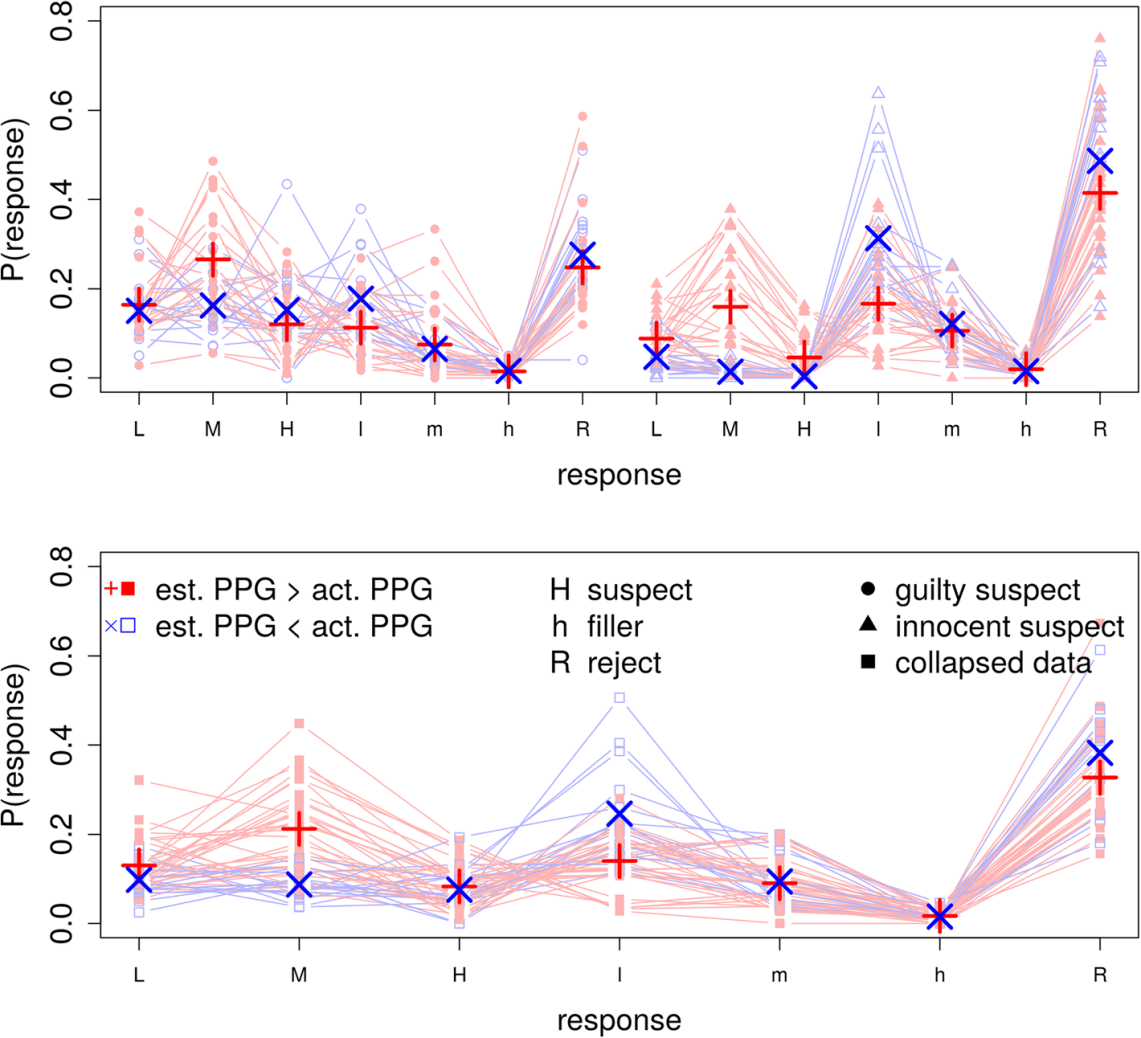
The response category proportions for all 47 experimental conditions with knowledge of the guilt or innocence of the suspect (top) or collapsed data (bottom). The lines show data from conditions in which the model-estimated PPG was higher (red, closed points) or lower (blue, open points) than the actual PPG. The larger symbols provide the means within each response category

So that the resulting misestimation of the base rates can be understood more easily, up to this point, we have highlighted the way in which the conditions of two specific studies were particularly challenging to the model. In turns out, however, that there is a systematic pattern in the data across all experimental conditions that predicts poor base-rate estimation. Figure 9 shows the distribution of identification responses for all 47 experimental conditions from Table 2. The blue points show the responses from all conditions in which PPG was underestimated. The red points show the results from all of the conditions in which PPG was overestimated. Although, for exposition, the data in the top panel of Fig. 9 are separated by innocent-suspect and guilty-suspect trials, recall that the model was fit to the collapsed data, which are shown in the bottom panel. The difference between medium-confidence suspect identifications (top panel, right-hand M) and the low-confidence filler identifications (top row panel, right-hand l) in innocent-suspect lineups accounts for 52% of the variability in the model PPG misspecifications (*r* = .72, *p* = .04). The reason that these particular response rates are challenging for the model is that fillers and innocent suspects are assumed to be sampled from the same distribution (see Fig. 2). This means that the model is forced to predict that the conditional distribution of confidence levels is the same for both of these lineup members; when the data show more identifications of innocent suspects than fillers, which tends to happen with moderate confidence, it resolves this conflict by incorrectly concluding that those suspect identifications are to guilty suspects and thus overestimates the base rate of lineups that include guilty suspects. In contrast, when the data show more filler identifications than innocent-suspect selections, the model resolves this conflict by concluding that there are fewer lineups containing guilty suspects and thus underestimates the base rate. A shift of criterion cannot completely account for this pattern because, for example, an increase in the medium-confidence response region will simultaneously increase the probably of medium-confidence suspect and filler responses, a pattern which does not occur. A similar pattern emerges for both the guilty-suspect trials and in the collapsed data.

#### Best practices

**Fig. 10:**
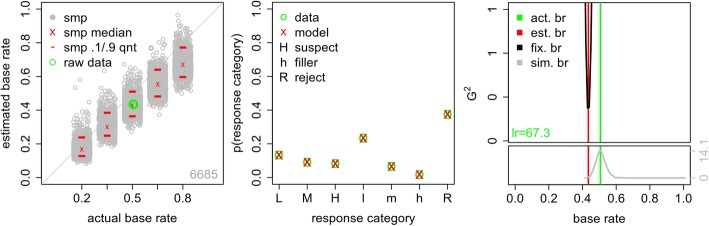
Results of the signal detection theory (SDT) model analysis when fit to the best-practices data set. Left: actual and estimated base rates when combined across all best-practices data. The green circle shows the actual experimental base rate and the SDT-model estimate. Each gray circle shows the estimated base rate for one of the sampled data sets (smp), with the sampled base-rate value jittered for visibility. The red lines and red x show the 10th and 90th quantiles and median of these estimated base rates. The number in the lower-right corner is the overall sample size. Middle: data results and model predictions for low- (L), medium- (M), and high- (H) confidence suspect identifications, low- (l), medium- (m), and high- (h) confidence filler identifications, and lineup rejections (R). Right: the green line shows the actual base rate in the experimental data and the red line shows the estimated base rate from the SDT model. In the upper section, the black curve shows how the model fit value changes as the model base rate varied. The number in the lower-left provides the likelihood ratio (lr) of the model when the estimated and actual experimental base rates are used. In the lower section, the gray curve shows the distribution of estimated base rates for data simulated from the SDT model with the model base rate fixed at the actual experimental base rate. The *y*-axis is frequency

**Fig. 11:**
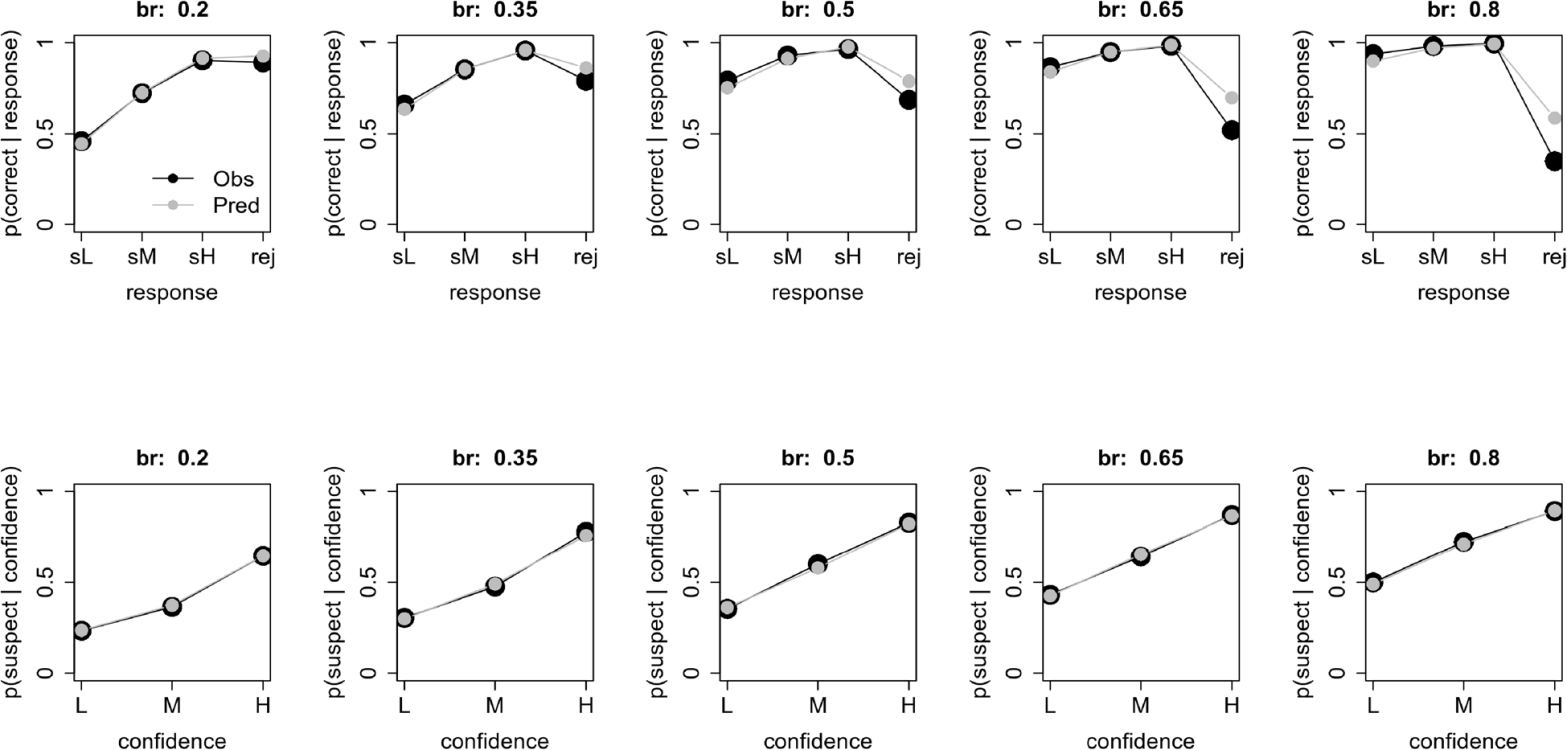
Calibration curves using the best-practices data set for both experimental data and model predictions. Each column is for a different sampled base rate. The top row plots the proportion of correct responses given either a rejection (rej) or a low (sL)-, medium (sM)-, or high (sH)-confidence subject response. The bottom row plots the probability of a suspect identification given a low- (L), medium- (M), or high- (H) confidence response (suspect or filler)

One of the strengths of the previous analyses is that they show how well the SDT model performs under a wide range of situations. Many of the experimental conditions, however, were specifically designed to deviate from best practices for lineups; for example, with the inclusion of biased instructions or fillers that are dissimilar from the suspect. Thus, they may not provide a good indication of how well the model would perform under ideal conditions (e.g., fair lineups, good witness instructions, double-blinded administration). Indeed, the previous section illustrates that the model fails to capture performance in exactly these problematic conditions. It is, therefore, informative to examine model performance when these less-than-ideal conditions are removed from the data set. We call the remaining data sets the *best-practices* data set. See the “Methods” section for details. We repeated our previous analyses on these data; the base-rate estimation, model fit, and sensitivity results are provided in Fig. 10, and the calibration curves are shown in Fig. 11.

**Fig. 12:**
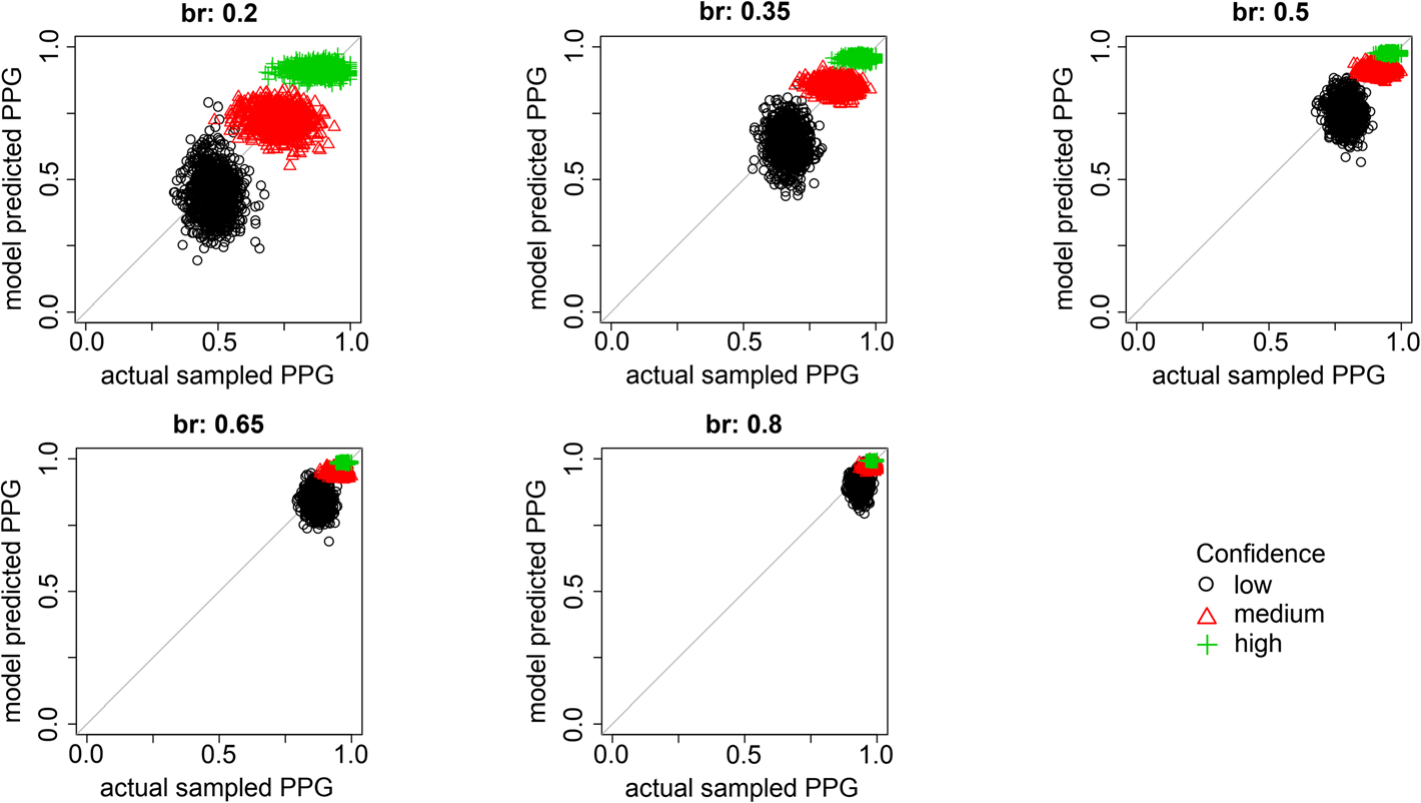
The model-predicted posterior probability of guilty (PPG) plotted against the actual sampled PPG for different base rates sampled from the best-practices data set, for low-, medium-, and high-confidence responses

The model fit is excellent. The bias to overestimate the proportion of guilty suspects for low base rates is now gone. Indeed, the model is very well calibrated at the lower end of the scale. There is, however, still a tendency to underestimate the proportion of guilty suspects at the higher end of the scale, and the estimates are still quite variable considering the large sample size (1000 witnesses). Furthermore, the deviations from the calibration curves are greatly reduced. The only exception is a tendency to overestimate the proportion of correctly rejected lineups, especially at high base rates. A comparison of the actual sampled PPG and model-predicted PPG is provided in Fig. 12. When restricted to these conditions, although there is still considerable variability across sampled data sets, the model does a very good to excellent job of estimating PPG at all base rates and all levels of confidence.

### Simulations

We have shown that the SDT model does a good job overall in estimating the true base rate of lineups that include the guilty suspect from collapsed data. Next, we consider the theoretical limits of this performance level using parameter recovery simulations. We generated a large number of data sets with the SDT model and then fit each simulated data set with the same model, allowing all five parameter values to vary freely (again, note that we fit an equal-variance model, *σ*_*g*_ = 1). At issue in this analysis is the ability of the SDT model to accurately recover the parameters that were used to generate the data. The generating parameters for each simulated data set were randomly sampled from highly variable distributions, so the model was challenged to accurately estimate the base-rate parameter, *p*_*g*_, against a background of random variation in all of the other model parameters. If the model succeeds, then we can conclude that there are data patterns that specify base rate in a way that cannot be mimicked by any combination of the other parameters. We used simulated data sets comprising 100, 500, or 1000 identification attempts to assess the effect of sample size. Full simulation details can be found in the “Methods” section. The code is provided on OSF.

**Fig. 13:**
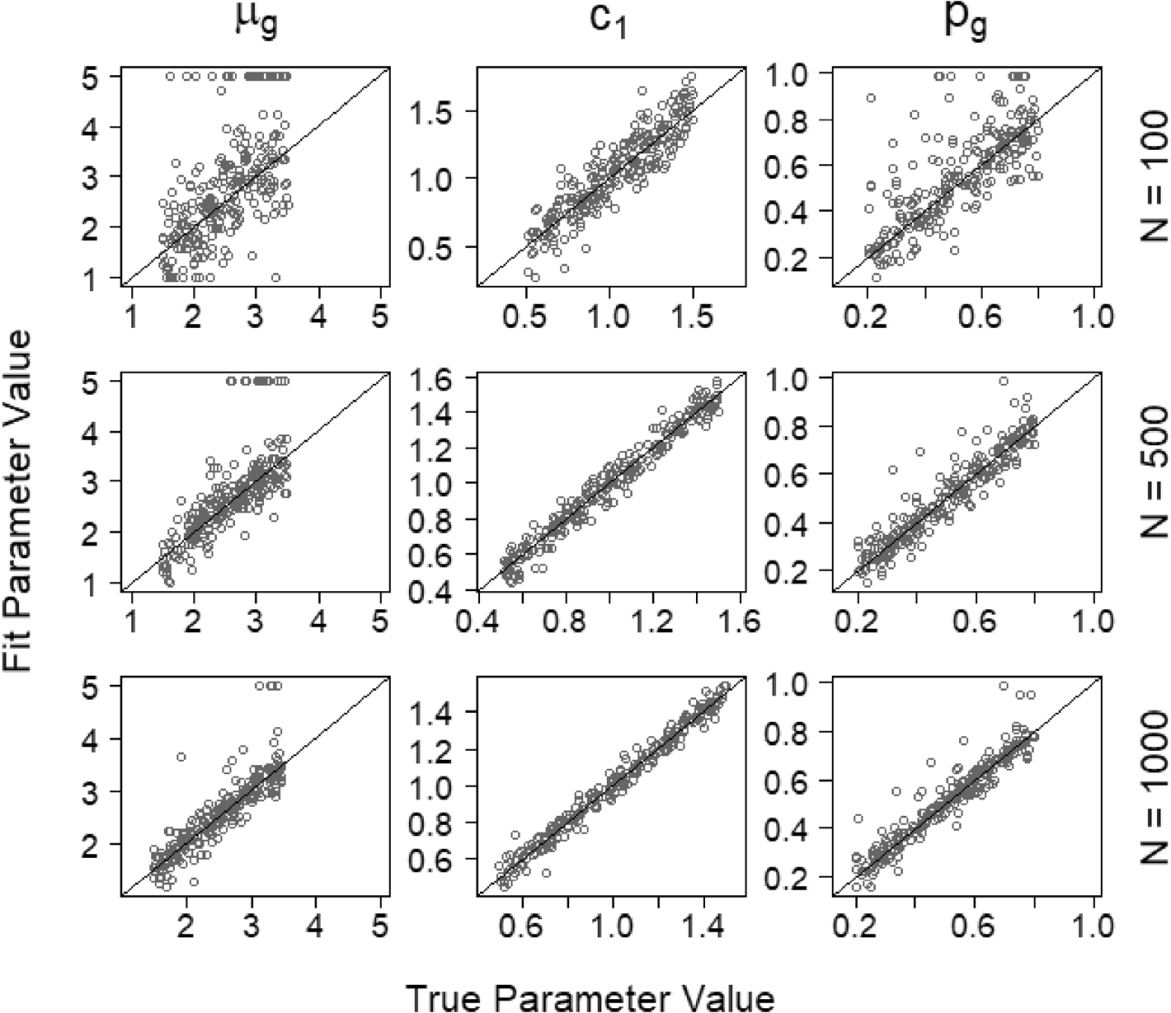
Parameter recovery of *μ*_g_, *c*_*1*_, and *p*_*g*_ from data simulated with the signal detection theory (SDT) model

Figure 13 shows the ability of the model to recover three key parameters: the distance between target- and lure-strength distributions (*μ*_g_), the identification criterion (*c*_*1*_), and the base rate of lineups with guilty suspects (*p*_*g*_). Each plot shows results for 250 fits to simulated data with the parameter value that generated the simulated data on the *x-*axis and the parameter value estimated in fits of those simulated data on the *y-*axis. In each case, the points tend to be concentrated along the positive diagonal, indicating accurate parameter recovery. Recovery for all parameters improves with larger samples, as expected. Notably, base-rate estimation is quite accurate with 1000 identification attempts per data set, with a strong majority of estimates within 5 percentage points of the true generating value. Our empirical results with 1000 identification attempts were noticeably more variable than the simulation results, suggesting that estimates based on real data are subject to additional uncertainty introduced by violations of the model’s assumptions, as discussed previously. Estimation sometimes failed for the *μ*_*g*_ parameter such that *μ*_*g*_ went to the maximum value allowed in the estimation program (which was arbitrarily set to 5). This failed estimation was most common at the smaller sample sizes.

The results in Fig. 13 show that the model is capable of distinguishing changes in base rate from changes in the other parameters, but it is not immediately obvious *how* the model does so. The latter question is especially mysterious given that collapsing the data seems to hide all information about base rates. We found that two aspects of the data are critical for this seemingly magical feat: the relative proportion of filler and suspect identifications and the distribution of responses across different confidence levels in each of these response categories. Although suspect identifications cannot be directly classified as correct or incorrect without knowing the guilt status of each suspect, filler identifications are known to be incorrect because the fillers are chosen from a pool of people known to be innocent. Thus, the model can gauge the extent to which things are “going well” – that is, a high proportion of suspects are guilty and witnesses are often successful in recognizing the true culprit – by evaluating the proportion of filler identifications (the lower the better).

**Fig. 14:**
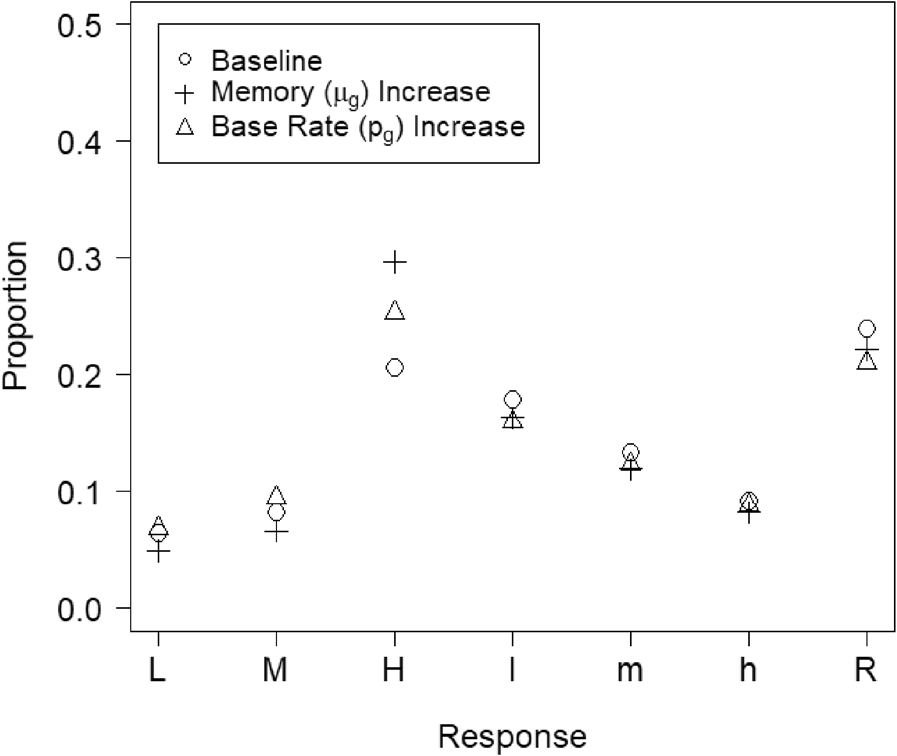
The change in the signal detection theory (SDT) response distribution collapsed across innocent-suspect and guilty-suspect lineups when the proportion of suspect picks is increased by changing either memory strength or the base rate of guilty suspects in the SDT model

How, then, does the model distinguish the two processes that might lead to troublingly high rates of filler selections, low base rate and poor witness memory? That is where the distribution across confidence comes in. Figure 14 demonstrates the role of confidence in estimating base rates. The circles are model predictions with a baseline parameter set, and the pluses and triangles are predictions generated by changing either the memory-strength parameter (*μ*_g_) or the base-rate parameter (*p*_*g*_), respectively, to increase the proportion of collapsed suspect identifications by the same amount. Boosting suspect identifications by improving memory strength also strongly increases the confidence for collapsed suspect identifications. The boost in collapsed suspect picks is produced because witnesses with a better memory are more likely to recognize guilty suspects in the subset of lineups that have them, and stronger memory also increases confidence in these guilty-suspect picks. Thus, increasing memory-strength results in fewer simulated suspect identifications being made with low and medium confidence. This effect occurs because the target-strength distribution shifts to the right with a higher *μ*_*g*_ value (i.e., increased memory strength), which also means that a greater proportion of this distribution falls in the highest confidence region. In contrast, increasing collapsed suspect identifications by increasing the base rate of guilty suspects has a flatter response profile across the confidence levels, showing a less dramatic increase in high-confidence suspect identifications compared to a memory change, and no decreases in responses made with medium or low confidence. In this scenario, the change in collapsed suspect picks is not driven by a change in responses to guilty *or* innocent suspects, but by a higher-level change in how these two trial types are proportionally mixed. Showing witnesses more lineups with guilty suspects means that there are more trials likely to produce a suspect identification, but does not mean that witnesses will be more confident when they do identify a guilty suspect.

Interestingly, the difference in response distributions due to changes in base rate and memory strength cannot be eliminated by allowing the confidence criteria to vary. The critical reason is that changing the confidence criteria also changes the confidence distribution for filler identifications. Figure 14 shows that the distinct confidence profiles for base rate and memory changes occur only for suspect selections and not filler identifications. Thus, changing the confidence criteria cannot make a base-rate change look like a shift in memory strength because the filler identification decisions would be distorted in detectable ways.

**Fig. 15:**
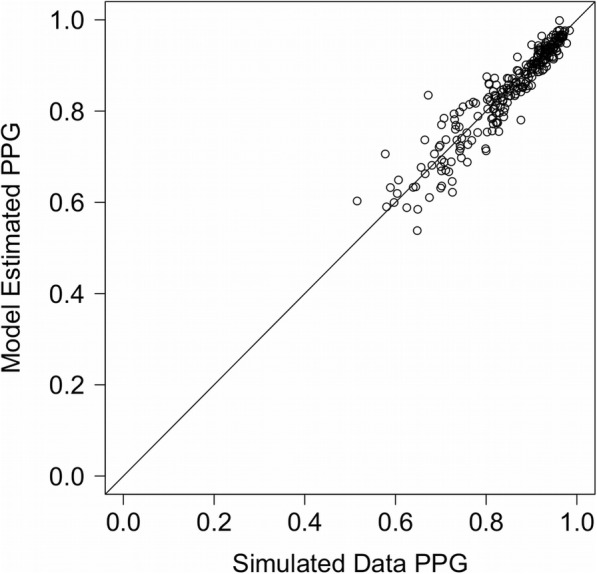
Actual and estimated posterior probability of guilt (PPG) for data simulated by the signal detection theory (SDT) model

We also used the simulations to assess the theoretical ability of the model to estimate PPG. Figure 15 shows a scatterplot of the actual PPG for each data set of 1000 simulated witnesses and the estimated PPG generated by fitting the model to each data set. Similar to the parameter recovery results, the points closely follow the positive diagonal, indicating excellent recovery of PPG. Again, these results are cleaner than the analyses that bootstrapped data from real data sets, suggesting that the analyses of real data are subject to additional uncertainty based on violations of model assumptions.

## Discussion

When analyzing real-world data from eyewitness identifications, it is, unfortunately, not possible to precisely determine how a witness identification of a suspect should change our belief in the guilt of the suspect. One problem is that the probability of guilt after the suspect has been identified, the PPG, can only be meaningfully estimated if we also know the proportion of lineups that include a guilty suspect, P(guilty).

Recent work (Wixted et al., 2016) successfully used a SDT model to estimate P(guilty) on a single empirical data set that provided the model with no information about the guilt or innocence of the suspects. The main goal of the current work was to extend this prior work by testing the ability of the SDT model to estimate P(guilty) on a much wider range of empirical data.

To test the SDT model, we collated data from 13 experiments and more than 10,000 identification decisions. The experiments varied in simulated crime, lineup sizes, the similarity relations of fillers and suspects, exposure and retention intervals, the position of the suspect in the lineup, different types of biases, the presence of a weapon, the use of alcohol by the witness, and the particular individuals used in the lineups. All experiments provided the lineup photos simultaneously and collected confidence ratings.

From these experiments, we generated data sets with a known proportion of guilty suspects. To mimic a real-world setting in which the guilt or innocence of the suspect is not known, the data were then collapsed over guilty-suspect and innocent-suspect trials. We then used the SDT model to estimate the base rate of guilty suspects from these collapsed data sets. We took advantage of new software and results, including closed-form solutions for the SDT model, that aid in fitting SDT models to lineup data (Cohen et al (n.d.) (in press)).

When tested on data drawn from this full data set, the model did a good job estimating base rate. When the actual base rate was low, however, both the estimated base rate and estimated PPG were too high. When the actual base rate was high, the estimated base rate was somewhat low. There was also considerable variability in these estimates. Estimation was excellent for base rates near 50%.

To get a sense of the generalizability of the results, the model was then applied to data from each of the 13 experiments separately. The results varied, with some experiments showing very good base-rate estimation and others showing extremely poor base-rate estimation. A closer look at individual experimental conditions demonstrated that conditions that bias participants to select the suspects too often or too seldom, for example, by manipulating the similarity of the suspect and fillers, are particularly troublesome. An analysis of the result patterns suggests that the model is misspecified under these conditions. The basic idea is that the biasing conditions tend to increase or decrease the proportion of suspect identifications. Critically, these changes are selective, so that the response pattern changes cannot be accounted for solely by shifts of response criteria. Thus, the model, in turn, interprets a shift toward or away from suspect identifications as a change in base rate. Indeed, when the model was applied to only conditions that approximated fair lineups, the best-practices data set, estimation of both base rates and PPG was very good to excellent, although high base rates were still slightly underestimated and variability in estimates was high given the large sample size. Further evidence that the model failure is due to a model misspecification comes from simulations of the SDT model which suggest that, when the data conform to the model assumptions, parameter estimation is very accurate. Estimation of both base rate and PPG was more accurate for model-generated data than empirical data, suggesting that the empirical data are subject to inaccuracies based on violations of the model’s assumptions.

One caveat should be noted, which is that this work assumes an equal-variance SDT model. That is, variability in the memory strengths of both targets and lures are assumed equal. This assumption is a mathematical requirement – without it, the model is under-constrained, and the base-rate parameter cannot be uniquely identified. In favor of this assumption, Wixted et al. (2016) found that an equal-variance SDT model was sufficient to account for both the uncollapsed and collapsed data from Palmer et al. (2013). It may be prudent, however, to treat the equal-variance assumption with skepticism. Indeed, a host of prior work suggests that the memory strengths for targets should be more variable than the memory strengths for lures (for a recent review, see Rotello, 2017). We acknowledge, however, that it is an open question whether and/or when the equal-variance assumption is violated in a lineup context. Regardless, a consequence of inappropriately assuming an equal-variance model is that measures of memory accuracy will be confounded with differences in witnesses’ tendency to choose from the lineup (Rotello, Masson, & Verde, 2008). For this reason, we currently recommend against using the model in this form to compare performance across different uncollapsed, experimental conditions.

To determine the effect of incorrectly assuming an equal-variance model, we fit an equal-variance SDT model, letting the base-rate parameter vary, to data simulated from an unequal-variance SDT model. In particular, we performed the same simulation as previously, but assumed that the standard deviation of the target distribution was 1.5, 50% higher than the lure standard deviation. Details are provided on OSF. The main result was that the individual parameter estimates were biased: Estimates of *p*_*g*_ were systematically low and estimates of *μ*_g_ were systematically high. It is interesting to speculate that the low base-rate estimation seen in some studies, such as Carlson et al. (2017), could be due to an incorrect assumption of equal variance. Remarkably, however, when estimating PPG, these biases cancelled each other out – estimates of PPG were excellent. Thus, although individual parameter estimates were biased, estimates of PPG were more robust. Further exploring the effect of such model misspecifications is an important avenue of future research.

Overall, depending on the crime scene circumstances and the lineup procedures – that is, both estimator and system variables – the results vary from very poor to very good base-rate estimation. Assuming that biased lineups do not account for a large proportion of real-world situations, the worst case scenario regarding base-rate estimation is probably similar to the results from the full data set (Fig. 3), which slightly overestimates base rate and substantially overestimates PPG when the actual base rates are low. If lineup quality is higher, i.e., more akin to the lineups represented in the best-practices data set, then base-rate estimation is very good across a wide range and estimation of PPG is excellent (Fig. 10).

## Conclusions

The probability that a suspect who has been identified as the culprit is actually guilty, the posterior probability of guilt (PPG), can only be meaningfully estimated if we know the proportion of lineups that include a guilty suspect, P(guilty) (Wells et al., 2015). In real-world settings, with real-world data from eyewitness identifications, this information is, unfortunately, not known. Previous work (Wixted et al., 2016) has shown that a signal detection theory (SDT) model can be used to estimate P(guilty), but the technique was validated with data from a single study. Here, we extended this prior work by testing the ability of the SDT model to estimate P(guilty) on a wide range of empirical data. In summary, the SDT model was able to successfully recover P(guilty) in experiments that conformed to the model’s assumptions, but failed, to varying extents, when the lineups were biased. For unbiased lineups, estimation of both P(guilty) and PPG were very good to excellent. For biased lineups, both P(Guilty) and PPG were overestimated because the suspect will be chosen too often and with confidence that is high, whereas fillers will be chosen too rarely. Thus, under good testing conditions, the SDT model provides valuable information about the evidentiary value of an eyewitness identification.

## Methods

In this section, we first describe the data used in the analyses and then the details of the SDT model and simulations.

### Data

The data used in the following analyses were selected to have the following characteristics. First, the data were from simultaneous lineups; that is, all individuals in the lineup were viewed by the participant at the same time. Second, there were at least six individuals in the lineup; in fact, all experiments considered here used either lineups of length six or eight. Third, confidence judgments were collected. Fourth, both guilty-suspect and innocent-suspect trials were included and the guilt or innocence of the suspect was known on every trial. Finally, the data were either available in published materials or were provided to us by the authors.

#### Full data set and individual experiments

The data from 13 experiments were used in the analyses, with a total of 10,137 trials. The experiment references are provided in Table 1. If we analyzed data from only a subset of experiments in a multi-experiment report, each experiment is listed separately. Conditions within that experiment were collapsed. In a later analysis, we separately analyze experimental conditions. The table also provides the labels used for the experiments in the figures, the experiment sample size, the number of individuals in the lineup, the number of confidence levels, and the factors manipulated in the experiment.

**Table 1:** The experimental data used in the analyses

Reference	Data subset	Label	***N***	Lineup size	Confidence levels	# confidence levels	Factors
Brewer and Wells (2006)	Single exp.	BW,2006	1200	8	0 to 100 by 10	11	Instruction fairness (biased, unbiased), foil similarity (low, high)
Carlson et al. (2016)	Single exp.	C+,2016	1415	6	0 to 1 by .1	11	Exposure duration (3 s, 10 s), weapon (present, absent)
Carlson, Dias, Weatherford, and Carlson (2017)	Single exp.	C+,2017	1234	6	0 to 100 by 10	11	Weapon (present, absent, concealed)
Gronlund, Carlson, Dailey, and Goodsell (2009)	Single exp. (simultaneous only)	G+,2009	1279	6	1 to 7 by 1	7	Video quality (good, poor), guilty strength (strong, weak), innocent strength (weak, strong), lineup fairness (unbiased, intermediate, biased), suspect position (2, 5)
Kneller and Harvey (2016)	Single exp.	KH,2016	120	6	1 to 7 by 1	7	Alcohol (control, placebo, mild intoxication)
Mickes (2015)	Exp. 1	M,2015,E1	302	6	0 to 100 by 10	11	–
Mickes (2015)	Exp. 2 (simultaneous only)	M,2015,E2	238	6	0 to 100 by 10	11	–
Mickes et al. (2017)	Single exp. (confidence ratings only)	M+,2017,E1	978	6	0–30 and 40 to 100 by 10	8	–
Palmer et al. (2013)	Exp. 1	P+,2013	908	8	0–20 and 30,100 by 20	5	Exposure duration (90 s, 5 s), retention interval (imm., 6–103 days)
Rotello, Guggenmos, and Isbell (2015)	Exp. 1 (simultaneous only)	R,E1	437	6	0 to 100 by 10	11	Suspect position (2, 5)
Rotello et al. (2015)	Exp. 2 (simultaneous only)	R,E2	521	6	0 to 100 by 10	11	Suspect position (2, 5)
Rotello et al. (2015)	Exp. 3 (simultaneous only)	R,E3	376	6	0 to 100 by 10	11	Suspect position (2, 5)
Wetmore et al. (2015)	Single exp. (simultaneous only)	W+,2015	1129	6	1 to 7 by 1	7	Retention interval (imm., 48 h), lineup fairness (biased, unbiased, other), innocence strength (weak, strong)

Notes: e*xp*. experiment, *imm.* immediate

Law enforcement officers do not typically ask witnesses to make an identification from a lineup lacking an actual suspect. For this reason, it is necessary to identify a suspect on every trial, even for innocent-suspect trials. Where available, we used the innocent-suspect identified by the authors. Where unavailable, either because an innocent suspect was not designated by the experimenters or because the data summary tables did not clearly identify that individual, an innocent suspect was randomly selected from the fillers on each trial.

Across these 13 experiments, different numbers of confidence levels were used. In contrast, our strategy for fitting the model to data assumes that all studies have the same number of confidence levels. To resolve this issue, we followed the basic idea from Wixted et al. (2016) and normalized confidence ratings to a 0–1 scale, assigning low, medium, and high confidence to normalized responses below 0.65, between 0.65 and 0.85, and above 0.85, respectively. To normalize the data, we divided the response confidence level rank minus 1 by the number of possible confidence levels for that study minus 1. The subtraction is necessary because confidence level ranks start at 1, but we wanted a normalized confidence scale that starts at 0. For example, assume that a study had seven confidence levels and, on a particular response, the confidence rating was 4. The normalized confidence for that response is (4 – 1)/(7 – 1) = 0.50 (the mid-point of that particular confidence scale), which, because it is below 0.65, is classified as a low-confidence response. In certain cases; for example, where there were few responses in a confidence range, the data provided by the researchers were collapsed across confidence levels. In such cases, we provide the collapsed ranges and compute the normalized confidence based on these collapsed ranges.

#### Experimental conditions

In a subsequent analysis, we separately examine conditions from these 13 experiments. The details of a subset of these conditions are provided in the “Results” section. In Table 2 we briefly list the conditions, sample size, and figure labels for each condition.

**Table 2:** The data analyzed by condition

Reference	Condition	Label	***N***
Brewer and Wells (2006)	Low fairness, neutral instructions	BW,2006,LN	300
	High fairness, neutral instructions	BW,2006,HN	300
	Low fairness, liberal instructions	BW,2006,LL	300
	High fairness, liberal instructions	BW,2006,HN	300
Carlson et al. (2016)	Shown weapon, short exposure	C+,2016,SS	337
	No weapon, long exposure	C+,2016,NL	368
	Shown weapon, long exposure^a^	C+,2106,SL	710
Carlson et al. (2017)	No weapon	C+,2017,N	409
	Concealed weapon	C+,2017,C	418
	Shown weapon	C+,2017,S	407
Gronlund et al. (2009)^b^	Fair lineup, innocent weak	G+,2009,FIW	317
	Intermediate bias, innocent weak	G+,2009,IIW	308
	Biased lineup, innocent weak	G+,2009,BIW	325
	Fair lineup, innocent strong	G+,2009,FIS	312
	Intermediate bias, innocent strong	G+,2009,IIS	320
	Biased lineup, innocent strong	G+,2009,BIS	336
	Fair lineup, guilty weak	G+,2009,FGW	320
	Intermediate bias, guilty weak	G+,2009,IGW	316
	Biased lineup, guilty weak	G+,2009,BGW	323
	Fair lineup, guilty strong	G+,2009,FGS	314
	Intermediate bias, guilty strong	G+,2009,IGS	310
	Biased lineup, guilty strong	G+,2009,BGS	336
Kneller and Harvey (2016)	No alcohol	KH,2016,N	40
	Alcohol	KH,2016,A	40
	Placebo	KH,2016,P	40
Palmer et al. (2013)	Short exposure, short retention	P+,2013,SS	253
	Short exposure, long retention	P+,2013,SL	218
	Long exposure, short retention	P+,2013,LS	219
	Long exposure, long retention	P+,2013,LL	218
Wetmore et al. (2015)^c^	Fair, innocent weak, short retention	W+,2015,FIWS	178
	Fair, innocent strong, short retention	W+,2015,FISS	101
	Biased, innocent weak, short retention	W+,2015,BIWS	184
	Biased, innocent strong, short retention	W+,2015,BISS	107
	Other^d^, innocent weak, short retention	W+,2015,OIWS	111
	Other, innocent strong, short retention	W+,2015,OISS	107
	Fair, innocent weak, long retention	W+,2015,FIWL	176
	Fair, innocent strong, long retention	W+,2015,FISL	115
	Biased, innocent weak, long retention	W+,2015,BIWL	153
	Biased, innocent strong, long retention	W+,2015,BISL	110
	Other, innocent weak, long retention	W+,2015,OIWL	108
	Other, innocent strong, long retention	W+,2015,OISL	113

Note: Only a subset of these conditions are discussed in the text. The full set of results is provided on OSF

^a^Includes both the 3-s and 10-s weapon view. The culprit’s face was seen for 10 s in both conditions

^b^To perform the analysis, both guilty-suspect and innocent-suspect trials are needed. Both strength levels of the innocent or guilty suspects that were not fixed were included. All levels of video quality and suspect position were used

^c^To perform the analysis, both guilty-suspect and innocent-suspect trials are needed. The innocent weak/strong suspects were paired with the guilty strong suspects

^d^Details are not provided in Wetmore et al. (2015) or in the data regarding these instructions, but they were designed to be similar to police instructions, so were included

#### Best practices

We also performed an analysis with only the experimental conditions that were designed to conform to best practices for police lineups; e.g., fair instructions. For this analysis, we only analyze published data, i.e., the Rotello et al. (2015) experiments are not considered. The data used in this analysis are as follows: Brewer and Wells (2006) fair instructions; Carlson et al. (2016, 2017) all data; Gronlund et al. (2009) removing biased lineups and lineups with strong innocent suspects; Kneller and Harvey (2016) all data; Mickes (2015) all data; Mickes et al. (2017) all data; Palmer et al. (2013) all data; Wetmore et al. (2015) removing biased lineups and trials with strong innocent suspects. These data included 6685 trials across the 10 experiments.

### SDT model

The signal detection model was implemented as described previously. Following Wixted et al. (2016), we used an equal-variance SDT model (*σ*_*g*_ = *σ*_*l*_ = 1). We assumed three confidence levels. There were five free parameters: the mean of the suspect/target distribution (*μ*_g_), the base rate of guilty suspects (*p*_*g*_ = P(guilty) from Eq. 1), and the three criteria (*c*_*1*_, *c*_*2*_, and *c*_*3*_)^1^ The mean and standard deviation of the lure/filler distribution were fixed at *μ*_f_ = 0 and *σ*_*f*_ = 1, respectively. The standard deviation of the suspect/target distribution was also fixed at *σ*_*g*_ = 1.

The basic idea of the model is as follows. Assume a lineup of length six. If the culprit is present, one sample is drawn from the target distribution and five are drawn from the lure distribution of Fig. 1. The sample with the highest strength, *h*, drives the response. If *h* < *c*_*1*_ the lineup is rejected. If *h* > *c*_*1*_ and the sample is from the target distribution, the culprit is selected. If *h* > *c*_*1*_ and the sample is from the lure distribution, a filler is selected. The response is of low, medium, and high confidence if *c*_*1*_ < *h* < *c*_*2*_, *c*_*2*_ < *h* < *c*_*3*_, *c*_*3*_ < *h*, respectively. If the culprit is not present, all six samples are drawn from the lure distribution, one of these lures is designated as the suspect, and the model proceeds in the same fashion.

Because we are interested in cases in which the guilt or innocence of the suspect is unknown, the model predictions are collapsed over guilty-suspect and innocent-suspect trials. For example, if the model predicts 10% of trials are high-confidence suspect responses to a guilty suspect and 2% of trials are high-confidence suspect responses to an innocent suspect , the model predictions will have 12% high-confidence suspect responses. The same process is repeated for all response categories. The data were collapsed in the same way.

For computational efficiency, the model was implemented using the closed-form equations (Cohen et al (n.d.) (in press); also see Wixted et al., 2018). The model was fit using the G^2^ fit measure to the seven data points from the collapsed data (suspect or filler at high/medium/low confidence or reject). Model predictions were not allowed to go below 0.001. Cells with no data were removed from the fit measure.

The model predictions depend, in part, on the lineup length. The experiments used here were selected to have at least six photos in the lineup; in fact, there were always either six or eight photos. Thus, when combined, the data set was comprised of experiments using lineups of different lengths. To combine the fit measure across experiments, the same set of parameters was used to predict the data at each lineup length separately and then these fit measures were combined by weighting them by the proportion of trials of each lineup length.

As a baseline, the model was first fit to the original, unmodified data (all 10,137 trials). To generate data sets with different base rates, the model was also fit to resampled data from the original data. To keep sample size constant across experiments, we fixed the number of sampled trials at 1000^2^ Assume a desired base rate of 0.20, that is, we want 20% of the trials to be guilty-suspect trials. From the original data, 20% (200) samples were drawn with replacement from the guilty-suspect trials and 80% (800) samples were drawn with replacement from the innocent-suspect trials. The model was then fit to these 1000 trials as described previously. To produce a distribution of estimated base-rate parameters, this process was repeated 1000 times. This entire process was then repeated for base rates of 0.20, 0.35, 0.50, 0.65, and 0.80. This range of base rates was selected to cover the likely range of actual base rates of police lineups. We deemed it unlikely that lineups would have base rates lower than 0.20 or higher than 0.80.

In addition to the model fit, we also computed the following measures, used to generate calibration curves, for both the data and model. First, we calculated the proportion of suspect identifications at each confidence level that were guilty (see Wixted et al., 2016). Although not always done in prior work, for completeness, we also computed the proportion of rejected lineups for which the suspect was innocent (e.g., Wells et al., 2015). Second, we calculated the proportion of suspect identifications at each confidence level, regardless of the guilt of the suspect. This was done for the data from each sampled base rate. The results were averaged across all sampled data sets.

We also conducted two analyses designed to explore how sensitive the SDT model was to the specific value of the base-rate parameter, *p*_*g*_. First, we assessed how well the SDT model fits the data when a particular base rate is assumed. Second, we assessed how well the SDT model can estimate base rates when the data are known to be generated by the model, assuring that the model assumptions are met. Details of, and results from, these analyses are provided in the “Results” section.

The model was first applied to the entire combined data set from the 13 experiments of Table 1. The model was then applied to the data from each individual experiment and then to the conditions listed in Table 2.

### Simulations

The simulation code is available on the project’s OSF page. Each loop of the simulation routine involved the following steps:

1. A random parameter set was generated by randomly sampling each parameter value from a uniform distribution. The ranges were as follows: 1.5 < *μ*_g_ < 3.5, 0.5 < *c*_*1*_ < 1.5, distance between *c*_*1*_ and *c*_*2*_, 0.2 < *Δc*_*2*_ < 0.4, distance between *c*_*2*_ and *c*_*3*_, 0.2 < *Δc*_*3*_ < 0.7, 0.2 < *p*_*g*_ < 0.8.
2. A simulated data set was created by randomly sampling *N* trials from the signal detection model with the sampled parameter values. Each simulated data set had *Np*_*g*_ lineups in which the culprit was present (“target-present”) and *N*(1 − *p*_*g*_) lineups in which the culprit was not present (“target-absent”), just like the data in our empirical bootstrap analyses. Thus, in both the simulations and our main analyses, we are treating base rates as a descriptive statistic that applies to a given data set. Generalizing to a new data set would involve additional uncertainty, which could be represented in simulations by sampling the number of target-present lineups from a binomial distribution parameterized by *N* and *p*_*g*_. For target-present lineups, a response was selected by randomly sampling five strength values from the lure distribution for fillers and one from the target distribution for the guilty suspect, selecting the highest of these strength values, and comparing this highest value to the criteria. The same process was used for target-absent lineups, except that the innocent-suspect strength was sampled from the lure distribution.
3. The model was fit to the simulated data set by minimizing G^2^. Fits always began with the same initial parameter values: *μ*_*g*_ = 2, *c*_*1*_ = 1, *c*_*2*_ = 1.5, *c*_*3*_ = 2, and *p*_*g*_ = .5.
4. The true (data-generating) and fit parameter values were saved, along with the G^2^ value.

## Data Availability

The data sets generated and/or analyzed during the current study, the scripts used to analyze them, and the full set of results are available in the following OSF repository: https://osf.io/3qz5n.

## Abbreviations

*c*_1_, *c*_2_, *c*_3_: SDT-model response criteria
Conf.: Confidence
Exp.: Experiment
Imm.: Immediate
*p*_*g*_: Probability of sampling from the target distribution, i.e., a guilty suspect, in the SDT model
PPG: Posterior probability of guilty
PPV: Positive predictive value
SDT: Signal detection theory
*Δc*_*2*_, *Δc*_*3*_: SDT distance between criteria *c*_1_ and *c*_2_ and between criteria *c*_2_ and *c*_3_
*μ*_f_ and *σ*_*f*_: Mean and standard deviation of the SDT-model lure or filler distribution
*μ*_g_ and *σ*_*g*_: Mean and standard deviation of the SDT-model target or guilty-suspect distribution

## Footnotes

1 The following ranges were put on the fit parameters: .01 < *p*_*g*_ < .99, 0 <*μ*_*g*_ < 3, − 3 < *c*_*1*_ < 6, and .01 < *Δc* < 3, where *Δc* is the distance between successive criteria.

2 Different sample sizes were tried. The qualitative results for sample sizes above about 100 were similar; thus, we just report a sample size of 1000 here. This is also very roughly the size of the Houston data set used in Wixted et al. (2016).
